# Overcoming evolved resistance to population-suppressing homing-based gene drives

**DOI:** 10.1038/s41598-017-02744-7

**Published:** 2017-06-19

**Authors:** John M. Marshall, Anna Buchman, Héctor M. Sánchez C., Omar S. Akbari

**Affiliations:** 1 Divisions of Biostatistics and Epidemiology, School of Public Health, University of California, Berkeley, CA 94720 USA; 2 Department of Entomology, Center for Disease Vector Research, and Institute for Integrative Genome Biology, University of California, Riverside, CA 92521 USA; 3 Bioinformatics Research Group, School of Medicine, Tecnológico de Monterrey, Estado de México, 52926 México, USA

## Abstract

The recent development of a CRISPR-Cas9-based homing system for the suppression of *Anopheles gambiae* is encouraging; however, with current designs, the slow emergence of homing-resistant alleles is expected to result in suppressed populations rapidly rebounding, as homing-resistant alleles have a significant fitness advantage over functional, population-suppressing homing alleles. To explore this concern, we develop a mathematical model to estimate tolerable rates of homing-resistant allele generation to suppress a wild population of a given size. Our results suggest that, to achieve meaningful population suppression, tolerable rates of resistance allele generation are orders of magnitude smaller than those observed for current designs for CRISPR-Cas9-based homing systems. To remedy this, we theoretically explore a homing system architecture in which guide RNAs (gRNAs) are multiplexed, increasing the effective homing rate and decreasing the effective resistant allele generation rate. Modeling results suggest that the size of the population that can be suppressed increases exponentially with the number of multiplexed gRNAs and that, with four multiplexed gRNAs, a mosquito species could potentially be suppressed on a continental scale. We also demonstrate successful proof-of-principle use of multiplexed ribozyme flanked gRNAs to induce mutations *in vivo* in *Drosophila melanogaster* – a strategy that could readily be adapted to engineer stable, homing-based drives in relevant organisms.

## Introduction

The concept of using homing-based gene drive systems to rapidly invade wild populations and spread effector genes (e.g. conferring pathogen resistance) or to suppress and eliminate populations was first suggested by Burt in 2003^1^. These systems have the remarkable ability to cheat during meiosis, enabling them to rapidly spread into a population even if they confer a fitness cost to their host (2, 3). They achieve this by encoding a sequence-specific nuclease that generates a double-stranded break at one or more specific target loci in a host’s genome, directly opposite the drive. To survive, the cell is forced to rapidly repair the DNA break using its endogenous DNA repair machinery. Repair of the break using the homology-directed repair (HDR) pathway, for instance, can result in the drive system being perfectly copied into its competing allele. When this occurs in a germline cell, it effectively converts a heterozygote into a homozygote, allowing the system to circumvent traditional Mendelian inheritance patterns and to drive into a population^2, 3^. The first decade following this proposition saw moderate progress in the development of homing-based drive systems in the African malaria vector, *Anopheles gambiae* ^2, 4, 5^. More recently, the development of the CRISPR-Cas9 system has unlocked enormous potential for this technology, with highly efficient drive systems being developed in quick succession to modify laboratory populations of *Drosophila melanogaster* ^6^, *Saccharomyces cerevisiae* ^7^, the Asian malaria vector, *Anopheles stephensi* ^8^, and the main African malaria vector, *An*. *gambiae* ^9^.

The homing-based drive systems developed using CRISPR-Cas9 have a number of highly desirable features: they are relatively straightforward to adapt to new target sequences and to port to other species, and the constructs engineered thus far have extremely high transmission rates, being inherited by 90–99% of the offspring of heterozygous parents^6–9^. However, these systems are not without their shortcomings. Firstly, the CRISPR-Cas9-based constructs engineered to date are associated with high fitness costs, although this is likely not an inherent problem with CRISPR-Cas9 systems, and secondly, the homing process has been shown to be highly error-prone, leading to the creation of mutant alleles within a few generations^8, 9^. A significant number of these mutant alleles consist of small deletions that preserve the reading frame and could represent homing-resistant alleles, i.e. mutant alleles that are not associated with a fitness cost^8, 9^. This latter shortcoming is of concern for population suppression strategies because homing-resistant alleles have a strong selective advantage over functional homing alleles that disrupt an essential gene, leading to suppressed populations rapidly rebounding.

Mutant alleles may be generated in several ways. For instance, they can emerge when the cell mends DNA damage at the target site using the non-homologous end-joining (NHEJ) pathway instead of HDR following drive-induced target site cleavage. Mutant alleles may also arise due to incomplete or imperfect copying during HDR. The CRISPR-Cas9 system is particularly vulnerable to this due to its large size – the system consists of promoters, the Cas9 gene, guide RNAs and, depending on the strategy being implemented, multiple effector genes and associated regulatory elements, all of which need to be perfectly copied during HDR to ensure spread into a population. Indeed, for the CRISPR-Cas9 homing construct engineered in *An*. *gambiae* ^9^, incomplete homing or internal deletion events were observed in 47% (15 out of 32) of screened organisms in which an errorless homing event was not observed. These were derived from a minimum of seven originating erroneous homing events, one of which produced a small deletion that preserved the reading frame and may be considered a homing-resistant allele. Homing-resistant alleles may also arise *de novo* via random target site mutagenesis, and some organisms may intrinsically be resistant to homing activity at a given site due to genetic variation within a species.

Therefore, while CRISPR-Cas9-based homing systems have enormous potential for the targeted engineering of populations, significant technical improvements are required if this technology is to be successfully implemented in the field^2, 10^. Here, we focus specifically on the issue of homing-resistant allele generation for population suppression homing systems. We largely ignore homing allele fitness costs in this analysis as we consider these to be surmountable through tailored engineering efforts – in one of the constructs engineered thus far, fitness costs seem to result from the transgene being inserted into an eye color gene^8^, and in another, due to the element copying itself to somatic as well as germline cells^9^, both of which we believe to be addressable. The impact of homing-resistant alleles on homing-based population replacement strategies has been described by Noble *et al*.^11^ along with a design strategy that selects against the resistant alleles; however, this solution does not apply to the population suppression systems that we explore here.

To address the impact of resistant alleles on homing-based population suppression systems, we develop a mathematical model to estimate the maximum tolerable resistant allele generation rates to achieve stable, long-term suppression for populations of various sizes. Our results suggest that, to achieve meaningful population suppression, tolerable rates of resistant allele generation are orders of magnitude lower than those observed for current CRISPR-Cas9-based homing systems. We describe how the required rates can be achieved by targeting multiple locations in a gene through guide RNA (gRNA) multiplexing^2, 3, 11, 12^. Furthermore, we demonstrate successful adaptation of a multiplexed ribozyme-gRNA-ribozyme (RGR) approach previously demonstrated in yeast^13^ and in mammalian tissue culture cells^13, 14^ to *in vivo* mutagenesis of target genes in *Drosophila melanogaster*, and discuss possible designs for, and challenges inherent in, the gRNA multiplexing approach for engineering stable, homing-based suppression gene drive systems. Finally, we explore the scale of population suppression that can be achieved by using this approach.

## Results

The homing population suppression system we explore here is based on that described by Hammond *et al*.^9^ in which the CRISPR-Cas9 system is designed to target a gene required for female fertility. This has the effect that females homozygous for the homing allele are infertile; however, heterozygous and wild-type females have at least one functional copy of the fertility gene and are fertile. For sufficiently high homing rates and small fitness costs, this system can spread into a population while it reduces population fertility, eventually leading to a population crash^1^. Hammond *et al*.^9^ describe three strains that they engineered with this design. We consider the most successful of these – construct 7280 – for which the transmission rate from heterozygotes was ~99%, and heterozygous females had their fitness reduced by 90.7%. Approximately half (~47%) of those who did not inherit a functional homing allele from a heterozygous parent inherited a copy with errors, and a seventh of the erroneous homing events that produced these alleles generated alleles that could be considered homing-resistant. Although the Hammond *et al*.^9^ construct was not proposed as a useable drive system, we consider its parameter values as a basis that future homing-based drive systems could build upon.

### Model framework

The framework used to model this system is described in the Materials and Methods; but in short, we denote the homing allele as “H”, the wild-type allele as “h”, and the homing-resistant allele (mutant allele with no associated fitness cost) as “R”. HH females are infertile, while all other genotypes are fertile. Hh males and females produce H gametes in the germline at a frequency equal to (1 + *e*)/2, where *e* denotes the efficiency of homing, or “homing rate”. Hh individuals also produce R gametes in the germline at a frequency equal to *ρ*/2, where *ρ* denotes the resistant allele generation rate. The remaining gametes produced by Hh individuals are wild-type and are produced at a frequency equal to (1 − *e* − *ρ*)/2. Females heterozygous for the homing allele have their fertility reduced by a fraction, *s*, while other genotypes are equally fertile (except for HH females, which are infertile). The crosses describing this system are shown in Supplementary Figure 1.

We use a discrete population, stochastic framework incorporating density-dependence at the larval stage to model this system. Our framework is modified from one previously used to examine the spread of homing endonuclease genes (HEGs) through populations of *An*. *gambiae* ^5^, the main malaria vector and the species in which the CRISPR-Cas9-based constructs were developed by Hammond *et al*.^9^. This framework incorporates the egg, larval, pupal and adult life stages. Generations are overlapping and adult females mate once, retaining the genetic material of the male they mate with for the duration of their adult life. Since we are modeling a population suppression system, a discrete, stochastic model is needed to capture the chance events that happen at low population sizes. This also enables the simulation of a population crash. Density-dependence at the larval stage is important to include as it captures the phenomenon in which more larvae survive to emergence when populations are small due to reduced larval competition. To model this, we consider a monotonic increase in larval mortality with larval density, as applied by Deredec *et al*.^5^.

### Expected dynamics of present constructs

**Figure 1:**
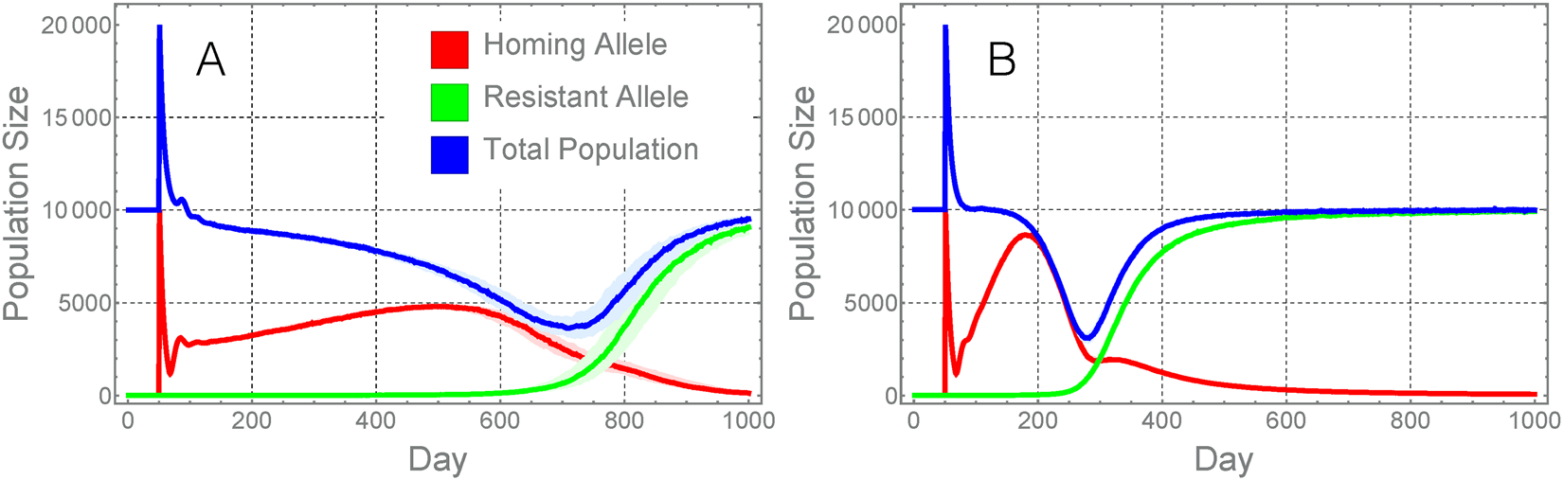
Predicted population dynamics for the present CRISPR-Cas9-based population suppression homing constructs. Here we model the predicted dynamics of the best-performing construct engineered by Hammond *et al*.^9^ in a population of 10,000 adult mosquitoes. The homing rate for this construct is ~98% and the resistant allele generation rate is ~0.13%. The model framework is described in the Materials and Methods. In panel (A), the dynamics are shown for the scenario in which females heterozygous for the homing allele have their fertility reduced by 90.7%. In panel (B), the same construct is modeled in the absence of a fertility cost. In both cases, population suppression is moderate and short-lived due to the generation of homing-resistant alleles leading to a population rebound. Red lines represent individuals having at least one copy of the homing allele (i.e. genotypes Hh, HR and HH), green lines represent individuals having at least one copy of the homing-resistant allele (i.e. genotypes hR, HR and RR), and blue lines represent the total population. Solid lines represent the median population size for 100 repetitions of the stochastic model, while shaded regions represent the 25–75% quartile range in these simulations, where this differs significantly from the median.

With the modeling framework established, we explore the predicted dynamics of construct 7280, the best-performing construct engineered by Hammond *et al*.^9^, in a population size of *N* = 10,000 adult mosquitoes. The results described in Fig. 1 correspond to a homing rate of *e* ≈ 98% (2 × (99–50%)) and a resistant allele generation rate of *ρ* ≈ 0.13% ((1 − *e*) × 15/32 × 1/7), i.e. of the wild-type alleles from heterozygotes that were not converted to homing alleles, 15/32 were mutant alleles and 1/7 of the events that produced these resulted in what may be considered homing-resistant alleles. The scenario in which females heterozygous for the homing allele have their fertility reduced by 90.7% is shown in Fig. 1A. Here, we see that gene drive occurs slowly and population suppression is moderate and transient. The total adult population falls by ~64% approximately one and three-quarter years following a 1:1 seeding release of HH males to hh males and females; however, this suppression is short-lived – a population reduction of 50% or more is only maintained for about five and a half months before the population rebounds.

Henceforth, let’s imagine that the fertility costs of the homing allele in heterozygous females can be prevented through engineering efforts to ensure that the CRISPR-Cas9 system is only expressed in germline cells. This scenario is shown in Fig. 1B. Here, we see that gene drive and population suppression occur more quickly, and the extent of population suppression is slightly greater (the population is suppressed by ~69% at its peak). However, the duration of suppression is very short – a population reduction of 50% or more is only maintained for about two months. This is far less than what is hoped for gene drive-based population suppression strategies, and is a consequence of the quick emergence of homing-resistant alleles once the gene drive system becomes prevalent in the population, leading to a population rebound.

### Design criteria for population elimination

**Figure 2:**
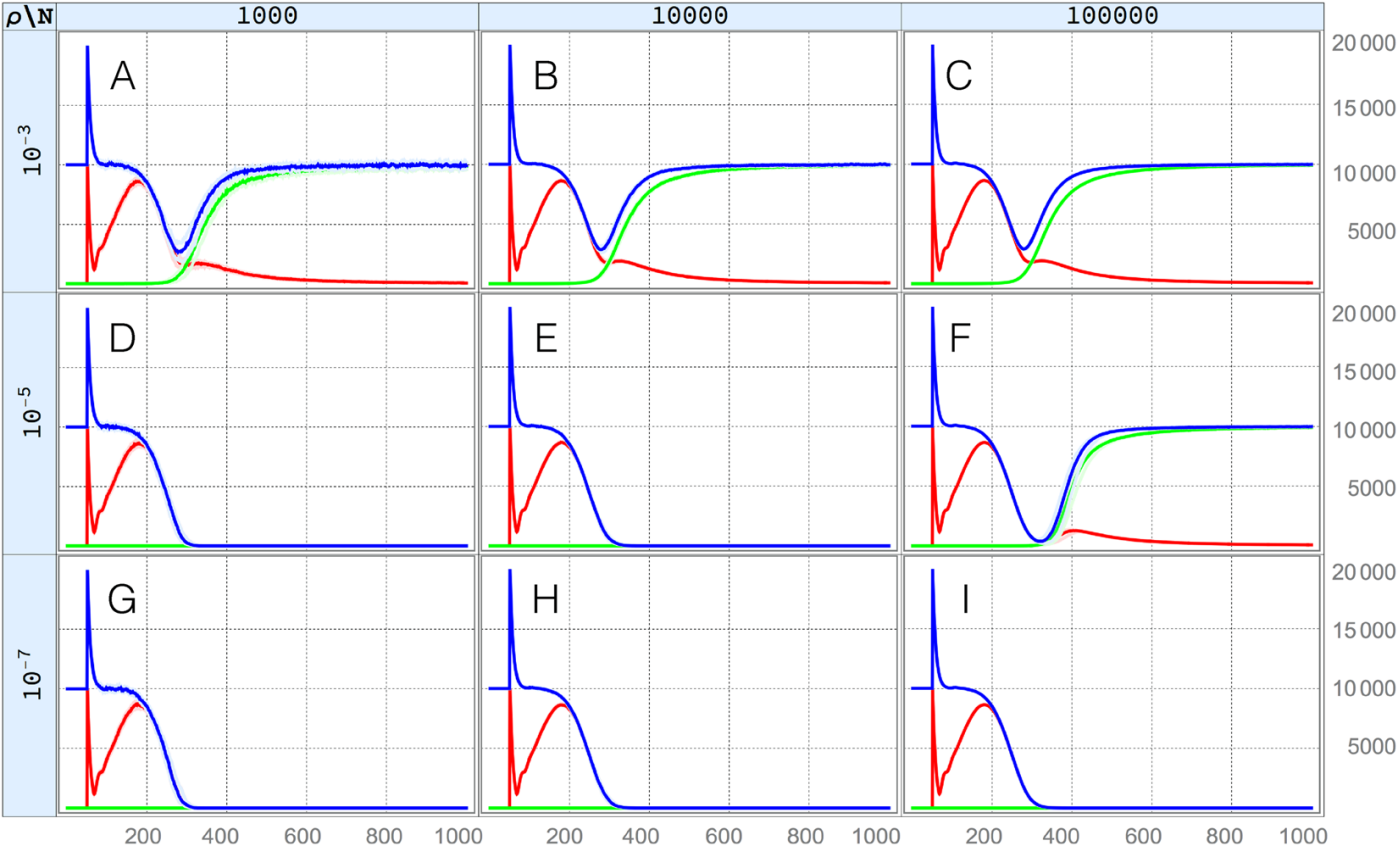
Homing and resistant allele trajectories for a range of population sizes and resistant allele generation rates. Here, we model a population suppression homing construct with a homing rate of 98% and no fertility cost. In panels (A–C) the resistant allele generation rate is 0.1% (10^−3^), in panels (D–F) it is 0.001% (10^−5^), and in panels (G–I) it is 0.00001% (10^−7^). In the leftmost panels (A,D and G), a population of 1,000 is modeled, in the middle panels (B,E and H), it is 10,000, and in the rightmost panels (C,F and I), it is 100,000. Red lines represent individuals having at least one copy of the homing allele, green lines represent individuals having at least one copy of the homing-resistant allele, and blue lines represent the total population. Solid lines represent the median value obtained from 100 repetitions of the stochastic model, while shaded regions represent the 25–75% quartile range, where this differs significantly from the median. As the resistant allele generation rate is reduced, we expect to eliminate populations of larger sizes.

The constructs engineered by Hammond *et al*.^9^ are clearly inadequate to lead to meaningful population suppression for population sizes of 10,000 adult mosquitoes; but presumably if the homing rate were increased and/or the resistant allele generation rate were decreased, then it may be possible to eliminate a specific population. In Fig. 2, simulations are shown in which homing efficiency is maintained at 98% while the resistant allele generation rate is reduced from 0.1% (10^−3^) to 0.001% (10^−5^) to 0.00001% (10^−7^) for populations of 1,000, 10,000 and 100,000 adult mosquitoes. Here, we see that, for a resistant allele generation rate of 10^−3^ (Fig. 2A–C), which is approximately what was observed for the Hammond *et al*.^9^ construct, we do not expect to be able to meaningfully suppress an adult population even as small as 1,000 individuals. However, if we reduce the resistant allele generation rate by two orders of magnitude to 10^−5^ (Fig. 2D–F), then we expect to eliminate populations of sizes 1,000 and 10,000, but not a size of 100,000. As the resistant allele generation rate is further reduced by an additional two orders of magnitude to 10^−7^ (Fig. 2G–I), we expect to eliminate adult populations of all sizes up to 100,000.

This trend of being able to eliminate populations of larger size with smaller resistant allele generation rates is intuitive as, in a larger population, there are more opportunities for error-prone homing events to occur, leading to the emergence of homing-resistant alleles. These resistant alleles will quickly be selected for, reversing any prior population suppression. The design target for the resistant allele generation rate will therefore be determined by the population size we wish to eliminate.

**Figure 3:**
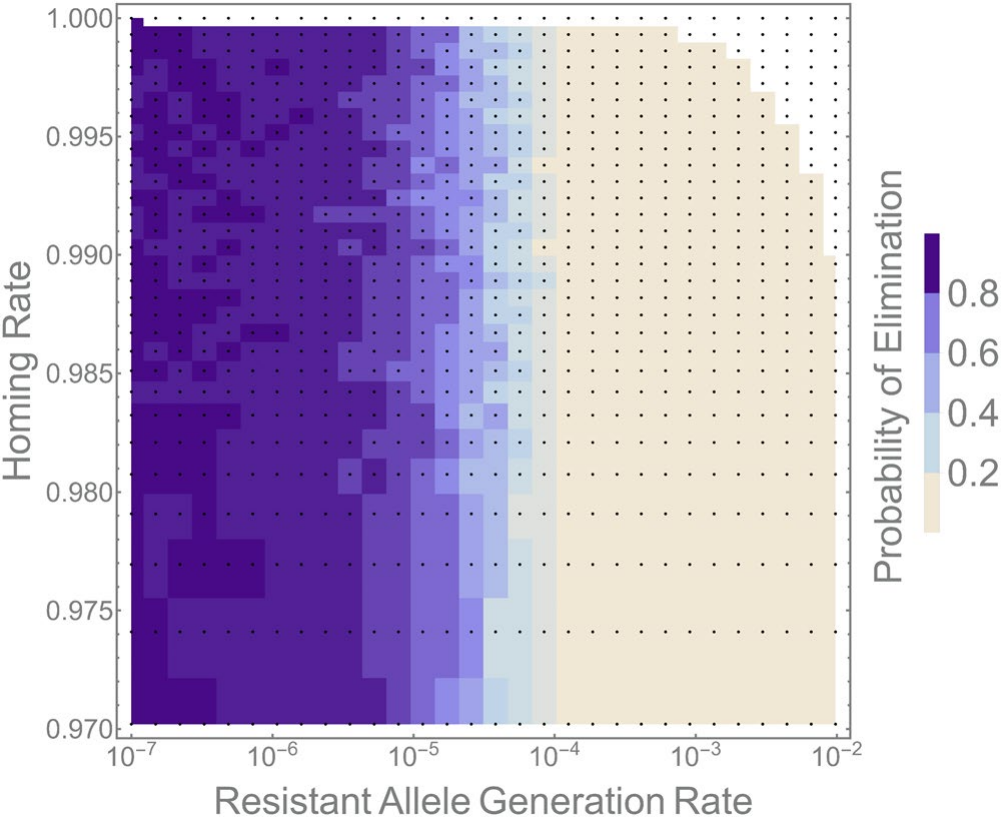
Dependence of population elimination probability on homing rate and resistant allele generation rate. Here, we model a population suppression homing construct in a population of 10,000 adult mosquitoes. Each pixel represents a combination of homing and resistant allele generation rates for which the simulation was run. Pixel shadings represent the proportion of 100 simulations in which population elimination was achieved within 950 days of a seeding 1:1 release of HH males to hh males and females. Both rate parameters were sampled logarithmically to gain higher resolution at high homing rates and low resistant allele generation rates. The white region represents impossible combinations of rate parameters (the rates would sum to >1). Population elimination probability is independent of the homing rate (for already high homing rates) and critically dependent on the resistant allele generation rate.

The homing rate, however, does not factor into these design considerations, at least for already high homing rates. Figure 3 shows the probability of population elimination as homing efficiency is varied between 97% and 99.99% and the resistant allele generation rate is varied between 10^−2^ and 10^−7^ for a population of 10,000 adult mosquitoes. The elimination probability is calculated as the proportion of simulations (from a total of 100 per parameter set) in which the *An*. *gambiae* population was eliminated within 950 days of a 1:1 release of HH males to hh males and females. Here we see that, for a population size of 10,000 adults, population elimination is highly likely for resistant allele generation rates smaller than 10^−5^, and is unlikely for rates above 10^−4^. There is a critical rate between these two values at which the population is equally likely to either rebound or be eliminated and, interestingly, these dynamics are independent of the homing rate for *e* > 98%.

### Dependence of the design criteria for ρ on N

**Figure 4:**
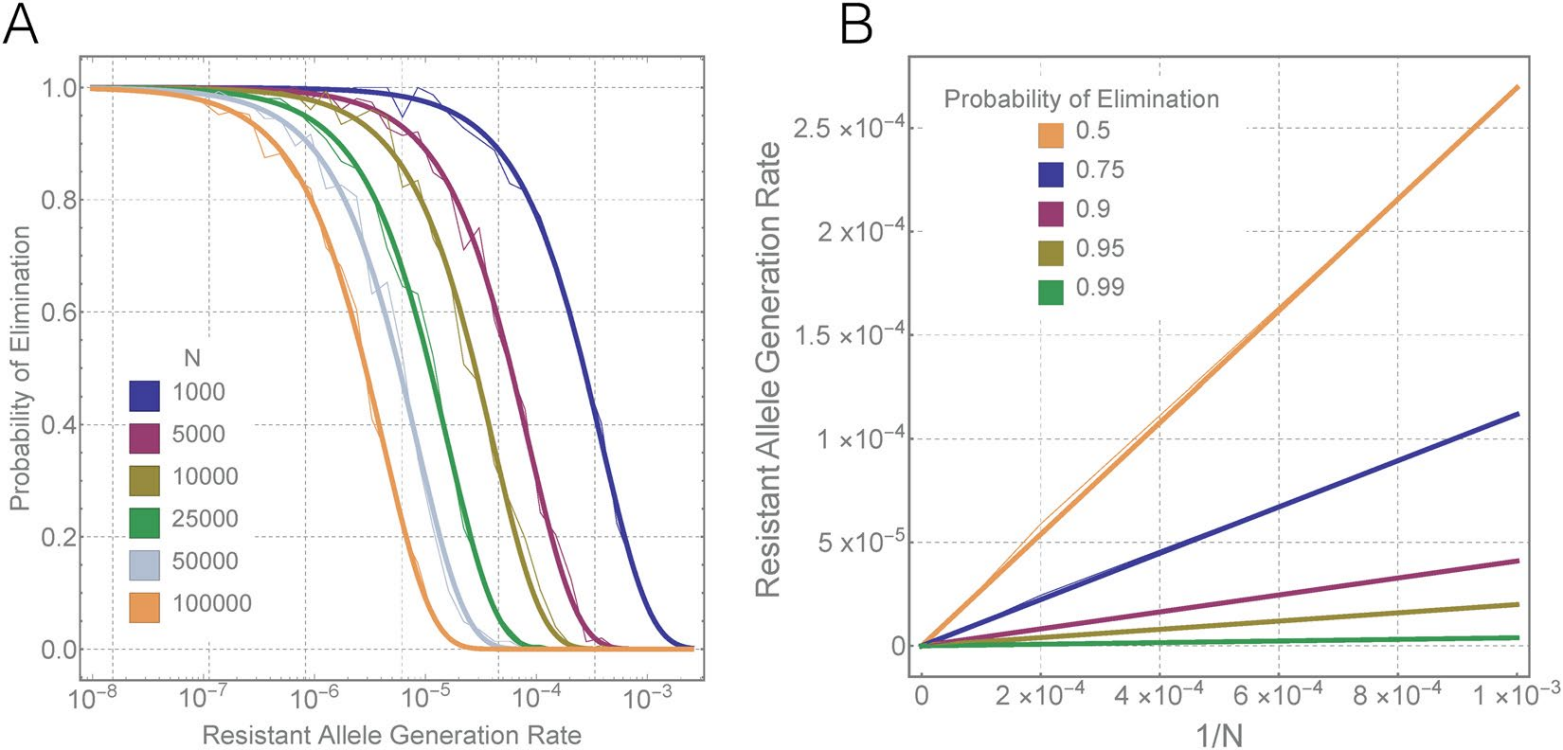
Relationship between population size and the resistant allele generation rate required for a given population elimination probability. (**A**) Elimination probability as a function of resistant allele generation rate for a range of population sizes, *N*, between 1,000 and 100,000. Sigmoidal curves are fitted to data points covering 30 resistant allele generation rates sampled logarithmically between 10^−2^ and 10^−7^. (**B**) Linear relationship between 1/*N* and the resistant allele generation rate leading to a given probability of population elimination. Values of 1/*N* are as shown in panel A, and resistant allele generation rates are inferred from the sigmoid curves. Faint lines in both panels represent interpolation between simulated data points while solid lines represent fitted linear relationships. There is a clear linear relationship between 1/*N* and the resistant allele generation rate leading to a given elimination probability.

The independence of elimination probability and homing rate (for already high homing rates) means that we can focus our attention on achieving a resistant allele generation rate, *ρ*, small enough such that population elimination is likely for a given population size, *N*. Stochastic simulations become highly computationally intensive as population size increases, and so we seek a relationship between *N* and the corresponding resistant allele generation rate, *ρ*, for which we can be 90% sure of achieving population elimination (or sure with some other degree of certainty). To this end, Fig. 4A depicts elimination probability as a function of *ρ* as we vary *N* between 1,000 and 100,000. The familiar case of a population size of 10,000 is shown in light gold and indicates that elimination is ~10% likely for a *ρ* value of 10^−4^ and ~80% likely for a *ρ* value of 10^−5^. As the population size increases from 1,000 to 100,000, we see that the *ρ* value required to achieve an elimination probability of 90% or higher becomes significantly smaller.

The form of the relationship between *N* and *ρ*_*x*_, the resistant allele generation rate leading to an elimination probability of *x*, is depicted in Fig. 4B for selected elimination probabilities. Fortunately for our ability to extrapolate to larger population sizes, there is a linear relationship between 1/*N* and *ρ*_*x*_ for all elimination probabilities investigated, i.e.:

1 $${\rho }_{x}={c}_{x}/N.$$

Here, *c*_*x*_ represents the slope from Fig. 4B corresponding to the elimination probability, *x*. To be 90% sure of population elimination, *c*_*x*_ is 0.0410, to be 95% sure, *c*_*x*_ is 0.0199, and to be 99% sure, *c*_*x*_ is 0.00391. This means that, to be 90% sure of eliminating a population of size 1,000, *ρ* should be less than 4.1 × 10^−5^ (0.0041%), and to be 90% sure of eliminating a population of size 100,000, *ρ* should be less than 4.1 × 10^−7^ (0.000041%). To be 95% sure of eliminating populations of these sizes, *ρ* should be about half that predicted for a 90% chance of elimination, and for a 99% chance of elimination, *ρ* should be an order of magnitude smaller than that predicted for a 90% chance of elimination. These estimates are derived for a mosquito population growth rate of *R*_*M*_ = 9.1 per generation, based on mosquito count data from the Garki project in Nigeria^15^. We repeated these analyses for low, medium and high population growth rates (*R*_*M*_ = 2, 6 and 12 per generation, respectively) and found that, while the slope, *c*_*x*_, changes, the linear relationship in Equation 1 still holds (Supplementary Figure 2).

The linear nature of the relationship between 1/*N* and *ρ*_*x*_ is understandable since each mosquito presents an opportunity for a resistant allele to emerge and prevent population elimination; however, it is reasonable to question whether population structure has a significant effect on the tolerable resistant allele generation rate. Supplementary Figure 3 explores the relationship between 1/*N* and *ρ*_*x*_ for randomly mixing populations of sizes 10,000–50,000 and for 1–5 randomly mixing populations each of size 10,000 that exchange migrants with the other populations at a rate of 1% per adult mosquito per generation. The linear relationship between 1/*N* and *ρ*_*x*_ is unchanged by the presence of population structure, which supports the intuition that each mosquito presents an opportunity for resistant allele emergence independent of population structure.

The population size that we wish to eliminate will vary depending on our goals; but one proposition for homing-based gene drive has been to eliminate a disease vector species such as *An*. *gambiae* on a continental scale. Assuming there are about ten times as many adult *An*. *gambiae* mosquitoes on the African continent as there are people, this suggests a population of ~10 billion (10^10^). The effective population size for a given elimination probability will be smaller than this due to population structure details and seasonal fluctuations in *An*. *gambaie* population size^16^; however, we consider this larger population size to provide conservative estimates of required *ρ* values. To be 90% sure that resistant alleles will not prevent elimination of a population this size, *ρ* should be less than 4.1 × 10^−12^ (0.0000000004%). To be 95% sure, *ρ* should be less than 2.0 × 10^−12^, and to be 99% sure, *ρ* should be less than 3.9 × 10^−13^.

### Multiplexing gRNAs

The *ρ* values required to prevent resistant alleles from interfering with the elimination of an *An*. *gambiae* population on the scale of the African continent are vanishingly small; but interestingly, the *ρ* value required to have a 90% chance of suppressing a population of just 1,000 adult mosquitoes is already several orders of magnitude smaller than that observed for the best-performing construct of Hammond *et al*. ^9^. Given the inevitability of the evolution of homing-resistant alleles, mitigating their impact is imperative to creating functional and stable homing-based population suppression gene drive systems.

**Figure 5:**
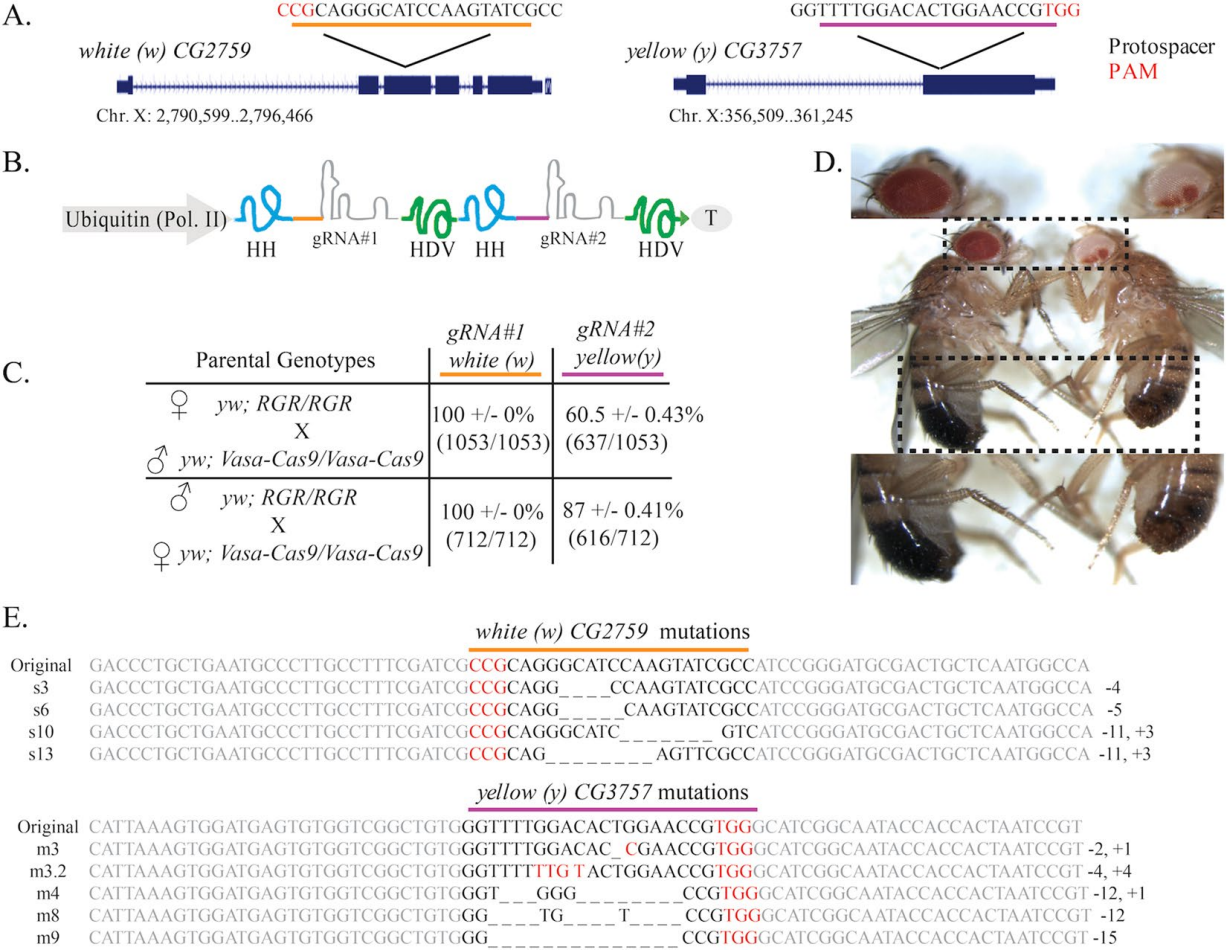
RGR/Cas9-induced mutations at the yellow and white loci. (**A**) Schematic of the white and yellow genes showing the gRNA target sites. Exons are shown as blue boxes, the gRNA target site locations are indicated by black lines, and the gRNA target site sequences (with black letters indicating protospacer sequences and red letters indicating PAM sequences) are underlined in yellow for white, purple for yellow. (**B**) Schematic of the OA-16 construct utilized in generating mutations. The first and second gRNAs (targeting white and yellow, respectively) are shown in grey. Each gRNA has a hammerhead ribozyme 5′ (shown in blue) and an HDV ribozyme 3′ (shown in green). The gRNAs are driven by a single *Drosophila* ubiquitin polII promoter. (**C**) Crossing scheme used to generate mutants, and obtained results. Individual male and female flies homozygous for the OA-16 construct were crossed to individual female and male flies, respectively, of a homozygous vasa-Cas9 line. Progeny were scored for eye and body color. Percentages correspond to number of flies out of total cross progeny (+/−SEM) exhibiting a mutation for each gRNA. (**D**) The white and yellow tissues of an OA-16 homozygous male fly with no exposure to Cas9 are un-mutated (left), while the white and yellow tissues of a fly generated by crossing OA-16 homozygotes to vasa-Cas9 homozygotes show mosaic expression (right). (**E**) Examples of sequences of CRISPR/Cas-induced mutations in white (top) and yellow (bottom). The first line in each alignment represents wild-type sequence, and subsequent lines show individual mutant clones.

A promising strategy for achieving this feat is the multiplexing of gRNAs in the gene drive to target multiple sequences. This idea has been previously proposed to increase the stability of drives^2, 3, 11, 17^; however, to date only one multiplexing strategy has been demonstrated to function in a whole-animal model^12^. Therefore, to further expand the toolbox for multiplexing gRNAs in whole animals, here we test the effectiveness of a technique previously demonstrated in yeast^13^ and in mammalian tissue culture cells^14^ that relies on flanking gRNAs with self-cleaving ribozymes, known as the ribozyme-gRNA-ribozyme (RGR) approach *in vivo* in *Drosophila melanogaster* ^13^. To validate the ability of this technique to induce mutations *in vivo* in *Drosophila*, we generated plasmid OA-16 that contains two multiplexed RGRs, the first targeting the *white* gene and the second targeting the *yellow* gene at target sequences previously validated^18^, driven by a single polymerase-2 ubiquitin promoter^19^ (Fig. 5A,B). The plasmid also contains a *white* gene that could be targeted by the *white* gRNA and used for detecting transgenic individuals bearing the OA-16 construct.

This plasmid was integrated site-specifically into the *Drosophila attP* line BSC (Bloomington Stock Center) 24486. Generated transgenic males and females were individually mated to females and males of transgenic line BSC 51324 (*vasa-Cas9*), and the progeny of the resulting crosses were scored (Fig. 5C). As expected, transformant flies bearing the OA-16 plasmid had no mutations in the *white* or *yellow* genes in the absence of Cas9 (Fig. 5D, left). All the scored offspring (712 from OA-16 male/Cas9 female crosses and 1053 from OA-16 female/Cas9 male crosses) had completely white or significantly variegated eyes (Fig. 5D), indicating that the first of the two RGRs had a relative somatic mutation efficiency near 100%, at least as measured by the proportion of individuals in which mutation events occurred. Although precise data were not compiled, mutation-bearing individuals gave rise to completely mutated, i.e., white-eyed, progeny at very high frequency, indicating functional germline transmission which is important for development of gene drives. Additionally, 87% +/− 0.41 (616/712) of the offspring of OA-16 male/Cas9 female crosses and 60.5% +/− 0.43 (637/1053) of the offspring from OA-16 female/Cas9 male crosses had a predominantly yellow cuticle (Fig. 5D, right), indicating that the second RGR was also functional, albeit with a significantly lower efficiency than the first. It should be noted that the efficiency of the second gRNA may be underestimated, as small mutant patches of yellow cuticle tissue are generally more difficult to detect than white patches of ommitidia facets in the compound eye. The presence of mutations was confirmed by sequencing of PCR products that span the cleavage site (Fig. 5E). Together, these data conclusively provide a proof-of-principle for the feasibility of the RGR approach as a method for multiplexing gRNAs in whole animals.

### Design requirements for multiplex number

This demonstration of the multiplexing of gRNAs is encouraging for the same being achieved in insect species that transmit human diseases, such as *An*. *gambiae*. Multiplexing is expected to increase the effective homing rate, as only one of several target sites must have a functional copy of the homing allele to be capable of homing. That said, as depicted in Fig. 3, the probability of population elimination is independent of the homing rate for *e* > 98%, but is highly dependent on the resistant allele generation rate, *ρ*. We derive the effective resistant allele generation rate for two and three multiplexed gRNAs in Supplementary Text S1 and find that this is approximately equal to *ρ* ^2^ and *ρ* ^3^, respectively. This logically follows since resistant allele generation in the presence of multiplexing requires all gRNA target sites to have a homing-resistant allele. In Supplementary Text S1 we show that, although homing-resistant alleles may accumulate in a composite allele with multiple target sites, partially resistant composite alleles are rarely generated and are frequently converted to homing alleles in subsequent generations. The rate of completely resistant composite alleles emerging is therefore approximately equal to the rate of resistant alleles emerging at all target sites at once, i.e. *ρ* ^*m*^ for a multiplex number of *m*.

The effective resistant allele generation rate therefore becomes exponentially smaller as the number of multiplexed gRNAs increases. For a baseline *ρ* value of 0.13%, the effective *ρ* value becomes ~1.7 × 10^−6^ for a multiplex number of two, and ~2.2 × 10^−9^ for a multiplex number of three. Following on from Equation 1, for a baseline resistant allele generation rate of *ρ* at a single site, the multiplex number, *m*, we must achieve to have a chance *x* of eliminating a population of size *N* is given by:

2 $$m > (\mathrm{log}\,{c}_{x}-\,\mathrm{log}\,N)/\,\mathrm{log}\,\rho .$$

This means that, to have a 90% chance of eliminating a population of size 10,000, we require a multiplex number of two; for a population of size 1 million, we require a multiplex number of three; and for a population of size 10 billion, we require a multiplex number of four. An encouraging property of these predictions is that, as multiplex number increases linearly, the population size that we can eliminate increases exponentially. A modest additional increase in multiplex number can also lead to a much higher chance of eliminating a population of the same size. For instance, a multiplex number of five is predicted to provide a > 99% chance of eliminating an *An*. *gambiae* population on the scale of the African continent. Important spatial factors are not considered here.

In the multiplexing experiments demonstrating the RGR approach in *D*. *melanogaster*, a reduced efficiency was observed at the second target site as compared to the first, for reasons that are unclear (but that may or may not be tied to position within the RGR array^12^). Presumably this is something that could be solved through subsequent engineering efforts; however, mathematical analysis described in Supplementary Text S1 shows that, at least for the two-gRNA system, a reduced cleavage rate at one site doesn’t significantly alter the effective resistant allele generation rate overall. This is because a reduced cleavage rate at one site also suggests a reduced resistant allele generation rate at this site, as both H and R alleles are generated through the same cleavage and repair mechanism. However, this reduction in the resistant allele generation rate is compensated for by the increased accumulation of partially-resistant composite alleles and their subsequent development into completely resistant composite alleles. The net effect is that, even if one of two multiplexed gRNAs displays a reduced cleavage rate, the benefits of multiplexing in terms of reduced resistant allele generation are very similar.

## Discussion

The possibility of using gene drive systems to suppress and potentially eliminate wild populations has provoked intense interest over the last decade^2, 3, 20–22^. This excitement has recently been fueled by significant developments in genetic engineering, and by the CRISPR revolution, which has enabled scientists to develop homing-based gene drive systems targeting a range of sites in any genome with relative ease. In terms of population suppression, recessive lethal and sterility genes are of interest as targets because a homing system targeting these genes can potentially spread to fixation and eliminate the population in the process, even when introduced beginning with a single drive-containing organism^4^. While this excitement is warranted, it is highly relevant to determine how the evolution of homing-resistant alleles could interfere with population suppression drive strategies, and to determine design criteria to increase stability of the drive for the likely elimination of populations of a given size.

To address this important question, we developed a mathematical model to describe the spread of a population-suppressing homing allele through a population of *An*. *gambiae*, and the impact that a homing-resistant allele could have on these dynamics. Homing-resistant alleles may originate through several mechanisms: a) *de novo* mutations, which occur independently from the drive; b) pre-existing natural variation in the population, which may be minimized through intelligent selection of homing recognition sites; and c) in response to the drive, either by errors introduced during HDR or by the cell mending DNA damage via the NHEJ pathway. While the former two mechanisms are important, we have focused this study on the latter (i.e. resistant allele formation in response to the drive), as this is expected to occur at a significantly higher frequency than the other two mechanisms^23^. Firstly, *de novo* mutations occur at a rate of ~10^−9^–10^−8^ per individual per generation in species similar to that of interest^24, 25^ – a rate that is many orders of magnitude smaller than resistant allele generation in response to the drive. Secondly, the frequency of resistance alleles in the population prior to the introduction of the drive system may be predicted based on mutation-selection-drift balance^26^. Unckless *et al*.^23^ have shown that pre-existing resistant alleles are likely when the effective population size exceeds 10^6^, a population size that would be several orders of magnitude higher for a composite allele. Furthermore, prior screening of target sites in wild populations could be used to identify pre-existing resistant alleles at frequencies less than 10^−3^, and at frequencies several orders of magnitude smaller for composite alleles. All of this highlights the significant relative importance of resistant allele generation in response to the drive.

We discover that, despite promising experimental data reporting extremely high rates of homing in the germline for recently engineered CRISPR-Cas9-based homing systems^6, 8, 9^, population suppression is expected to be moderate and short-lived with current construct architectures due to the generation of homing-resistant alleles. To minimize chances of a population rebound, current homing rates are adequate; however, reducing the resistant allele generation rate is crucial. For example, to be 95% sure that resistant alleles will not interfere with suppression of a population of *An*. *gambiae* mosquitoes on the scale of the African continent, the resistant allele generation rate should be less than ~2 × 10^−12^ per homing event – about nine orders of magnitude smaller than current resistant allele generation rates^9^.

While it might be near impossible to achieve resistant allele generation rates this low with a single gRNA recognizing an exclusive target site, one strategy to mitigate the impact of homing-resistant alleles is to multiplex gRNAs in the drive system^2, 3, 9, 17^. By multiplexing gRNAs to target multiple locations within an essential gene, each site is required to be homing-resistant in order for the composite allele to have the homing-resistant phenotype. Our results suggest that the effective resistant allele generation rate becomes exponentially smaller as the number of multiplexed gRNAs increases, and that a multiplex number of four may be sufficient to have a 90% chance of eliminating an *An*. *gambiae* population on the scale of the African continent.

**Figure 6:**
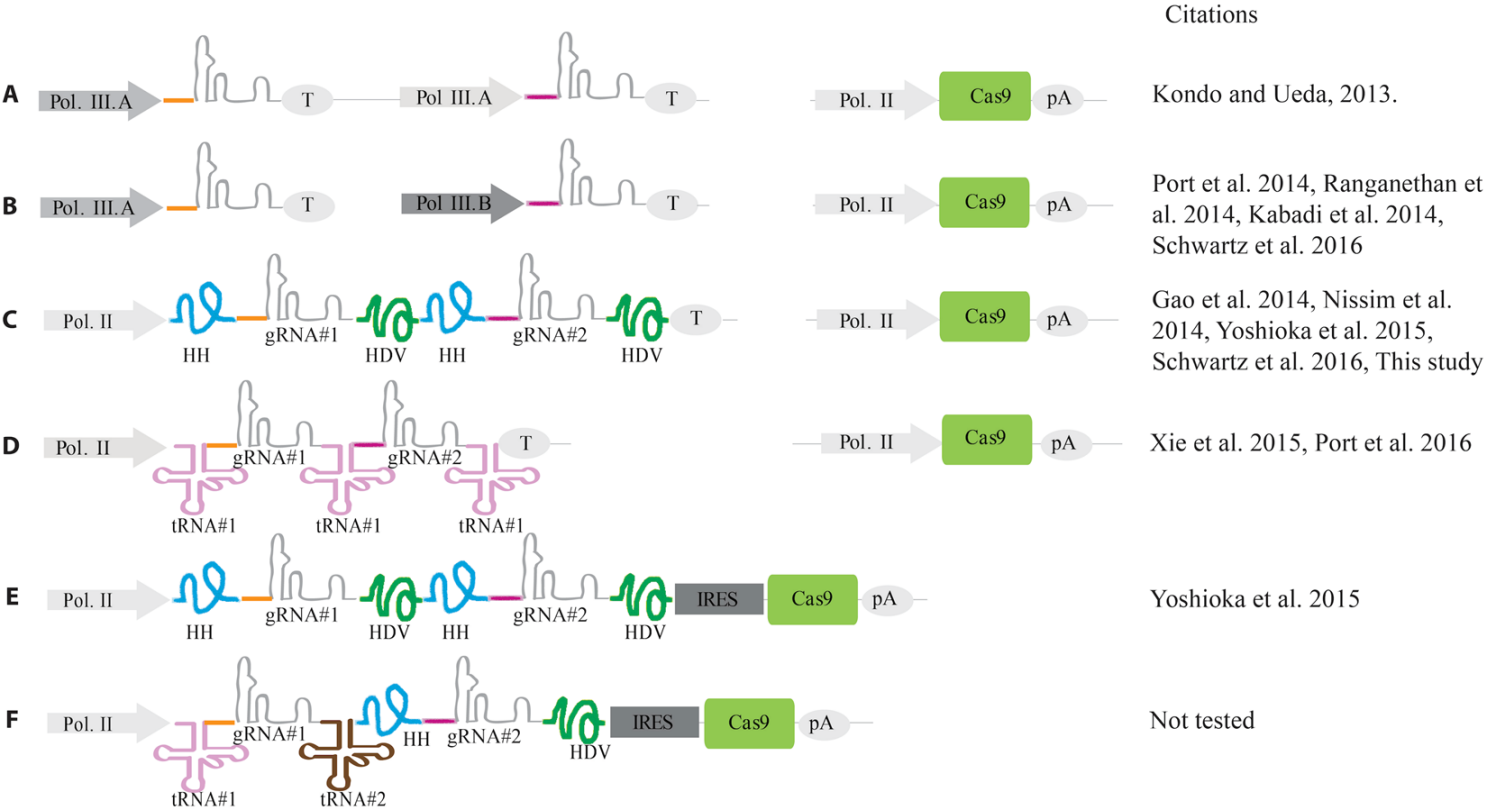
Schematic of various proposed strategies for multiplexing gRNAs. (**A**) A gRNA multiplexing scheme where the same polIII promoter drives each of two gRNAs, and a polII-driven Cas9 is provided as a separate transgene. (**B**) A multiplexing scheme where two different polIII promoter drive each of two gRNAs, and a polII-driven Cas9 is provided as a separate transgene. (**C**) A multiplexing scheme where each of two gRNAs are surrounded by a 5′ HH ribozyme and a 3′ HDV ribozyme, and a polII-driven Cas9 is provided as a separate transgene. (**D**) A multiplexing scheme where the two gRNAs are surrounded by copies of the same tRNA (with a tRNA 5′ of the first gRNA, between gRNAs 1 and 2, and 3′ of the second gRNA), and a polII-driven Cas9 is provided as a separate transgene. (**E**) A multiplexing scheme where each of two gRNAs are surrounded by a 5′ HH ribozyme and a 3′ HDV ribozyme, as in (**C**), but the Cas9 is located on the same transgene, 3′ of the gRNAs and preceded by an IRES. (**F**) A proposed multiplexing scheme where the first of two gRNAs is surrounded by two different tRNAs, the second gRNA is flanked by the HH and HDV ribozymes (as in (C) and (**E**)), and the Cas9 is located on the same transgene, 3′ of the gRNAs and preceded by an IRES (as in (**E**)). Grey triangles represent polIII promoters; blue triangles are polII promoters; terminators (T) and polyA signals (pA) are shown in grey ovals; Cas9 is represented as a green rectangle; internal ribosomal entry sequences (IRES) are grey sequences; gRNA scaffolds are shown as grey lines, with red and purple connecting lines representing two different gRNAs; the hammerhead (HH) and HDV ribozymes are shown as blue and green lines, respectively; and two different tRNAs (tRNA^Gly^ and a non-specific tRNA) are shown as pink and brown lines, respectively.

Several approaches to multiplexing gRNAs have been described, including the use of different polIII promoters such as U6:1-U6:3^27^, HP1^28, 29^, 7SK^28^, or tRNA promoters^30^ to promote expression of individual gRNAs (Fig. 6A,B). While these strategies are effective, they are limited by the fact that most polIII promoters do not drive temporal and/or tissue-specific expression, which may incur increased fitness costs to the organism due to ubiquitous and continuous gRNA expression. These strategies also require an individual promoter element for each gRNA, thereby increasing the overall size of the drive and possibly introducing repetitive elements. Repetitive DNA sequences have reduced stability^31^ and previous attempts to build drives with zinc-finger nucleases and TALENs have indeed demonstrated that larger and more repetitive the drive systems are less evolutionarily stable. Therefore, it is essential to minimize the size and repetitiveness of the drive^32^. To circumvent the need to express each gRNA from a different polIII promoter, gRNAs can be flanked with self-cleaving ribozymes^13, 14, 30, 33^ or tRNAs^12, 27, 30, 34^, which can allow the use of a single temporal and tissue-specific polII promoter to drive expression of an array of flanked gRNAs, thus reducing the overall drive size and repetitiveness of the drive element.

To date, only the tRNAflanked gRNA multiplex approach has been validated in a whole animal model^12^. Here, we report that the adaptation of another multiplex RGR approach, previously demonstrated in yeast^13^ and in mammalian tissue culture cells^14^, functions efficiently in *D*. *melanogaster*, at least with respect to the ability to be processed somatically and induce mutations via NHEJ. We observe highly efficient somatic mutation rates (as measured by the percentage of individuals possessing mutations) approaching 100% for the first gRNA and of 60%-86% for the second gRNA, depending on whether Cas9 is either maternally or paternally inherited (Fig. 5), and a high frequency of mutation transmission to the offspring of mutant individuals. While these observations do not directly indicate that the RGR approach will effectively function to induce HDR in the germline, they at least indicate that ribozyme-flanked gRNAs are processed *in vivo* and can direct efficient mutagenesis. Importantly, while we use the same two ribozymes to flank each gRNA (Figs 5B and 6C), the RGR approach may be expanded in the future to incorporate different ribozymes than the two tested here^35, 36^ to reduce repetitiveness, and the same strategy could be applied to the use of tRNAs other than the tRNA^Gly^ (Fig. 6D) that was shown to work by Port *et al*.^12^. Furthermore, as recently demonstrated by Yoshioka *et al*.^14^ in mammalian cells, the RGR approach could also be optimized to allow for expression of both the multiplexed gRNAs and CRISPR-Cas9 components from a single polII promoter, further reducing drive size and repetitiveness (Fig. 6E). Utilization of this single promoter-gRNA-CRISPR/Cas9 strategy combined with both the tRNA and RGR approaches may yield an optimal drive element design, both in terms of efficiency and stability (Fig. 6F). Finally, it may be important to utilize improved gRNA backbones to further reduce repetitiveness of the gRNAs^37^. Overall, the above design considerations may offer opportunities to engineer compact, evolutionarily stable gene drive cassettes; however, these ideas are largely untested and the use of multiplexed gRNAs in a functional gene drive system remains to be demonstrated.

Several assumptions have been made in the modeling portion of this study. Most noteworthy is the description of an *An*. *gambiae* population on the scale of the African continent as randomly mixing. Clearly, the study of gene drive in *An*. *gambiae* at anything beyond the village scale will require an understanding of population structure, and in fact, even at the village scale there are population considerations regarding gene flow within the *An*. *gambiae* species complex^38, 39^. By ignoring population structure, the model described here cannot be used to predict the spatial pattern or timescale of gene drive^40^. However, even in the presence of population structure, each target site on a chromosome represents an opportunity for homing resistance to emerge and, in this sense, we expect there to be some validity to predictions regarding the resistant allele generation rate required to ensure that a population rebound unlikely (Supplementary Figure 3). Recent work by Eckhoff *et al*.^41^ has modeled homing-based gene drive systems in a spatially-explicit manner with seasonality based on Namawala, Tanzania and the Garki District, Nigeria. For a seasonal population size of 10^3^–10^5^ mosquitoes, a critical NHEJ rate of 10^−5^ was observed, which is in agreement with our model^41^. One potential impact of population structure is the emergence of resistance to one guide RNA in one population and to another guide RNA in another population rarely combining due to low rates of gene flow. However, the suggestion in Supplementary Text S1 that partially resistant composite alleles are rare due to their low rate of emergence and quick conversion to functional homing alleles would argue against this concern.

At the molecular level, several assumptions have been made regarding the dynamics of multiplexed gRNAs. These have been modeled as independently-acting homing systems; however, the use of multiplexed gRNAs in a functional gene drive system has yet to be demonstrated and hence these dynamics will be elucidated in future drive experiments. Potential problems may arise from sequence repetitiveness in the drive element if identical gRNA backbones and promoters are used (Fig. 6), creating the possibility of recombination between identical sequences^12^ and thus reducing the overall evolutionary stability of the system^42^. Furthermore, it is not clear how the cleavage and homing rates will vary as multiplex number is increased substantially. If the gRNA target sites are far away from each other (e.g., >1–5 kb), then it is theoretically possible that multiplexing may not be an effective strategy as homology arms may not be able to effectively direct HDR (Supplementary Figure 4A–D), while if they are close together (e.g., <1 kb), multiplexing may increase homing effectiveness (Supplementary Figure 4E–H), as homology arms may able to effectively direct HDR, although this hypothesis remains to be demonstrated. Interestingly, a reduction in the homing rate associated with one of the gRNAs may not interfere with our design criteria for population suppression; however, it is important that we have a good quantitative understanding of the underlying molecular dynamics of multiplexed gRNAs to make accurate model predictions.

In conclusion, multiplexing gRNAs appears to be a highly effective strategy by which to reduce the effective resistant allele generation rate and hence to enable the elimination of large populations. Due to the exponential decrease in resistant allele generation with increasing multiplex number, only a modest number of gRNAs are needed to achieve population suppression potentially on a continental scale. These approaches need to be tested in relevant organisms to accurately describe their dynamics and to confirm their utility for population suppression strategies. Future studies should address additional sources of resistant alleles, such as *de novo* mutations and naturally-occurring genetic variation. Additional strategies for overcoming resistance should also be explored, for instance, engineering successive gene drive systems, each designed to target different essential genes, and releasing these one after the other^1^. While both this approach and gRNA multiplexing may be effective for overcoming resistance, neither has been demonstrated. Given how quickly this field is advancing, understanding strategies such as these should be of high priority so that the full potential of homing-based population suppression drives can be properly evaluated.

## Materials and Methods

### Modeling CRISPR-Cas9 population genetics

To characterize the basic dynamics of the autosomal CRISPR-Cas9-based gene drive system targeting a gene required for female fertility^9^, we represent the CRISPR homing construct as a single autosomal allele, “H”, with a corresponding wild-type allele, “h”. We denote homing-resistant alleles (mutant alleles with no associated fitness cost) as “R”. The CRISPR construct creates a bias among gametes of both parental heterozygotes towards gametes having the CRISPR allele. We consider the case where the homing rate is the same among Hh males and females and denote this as *e*. We also consider a resistant allele generation rate that is identical among Hh males and females and denote this as *ρ*. The homing rate, *e*, is the proportion of h gametes in heterozygotes that become H gametes due to the act of homing, and hence the proportion of H gametes arising from heterozygotes of both sexes is equal to (1 + *e*)/2. The resistant allele generation rate, *ρ*, is the proportion of h gametes that become R gametes due to errors introduced during the DNA breakage and repair process, and hence the proportion of R gametes arising from heterozygotes of both sexes is equal to *ρ*/2. The remaining gametes arising from heterozygotes are wild-type, h. This leads to an increase in the frequency of the H allele in the population and, since HH females are infertile, there is potential for a population crash to occur under permissive conditions. However, since the R allele represents resistance to homing and hence resistance to the spread of the H allele, it has a selective advantage following emergence and is expected to reverse the effects of population suppression. The crosses describing this system are shown in Supplementary Figure 1 and effective resistant allele generation rates for higher multiplex numbers are derived in Supplementary Text S1.

### Modeling An. gambiae population dynamics

Using *An*. *gambiae* as a case study, we adapt the modeling framework of Deredec *et al*.^4^ to describe the spread of the CRISPR and homing-resistant alleles through a discrete, density-dependent population with time steps of one day. In this model, the mosquito life cycle is divided into four life stages – egg, larva, pupa and adult (both male and female adults are modeled). The daily, density-independent mortality rates for the juvenile stages are assumed to be identical and are chosen for consistency with the population growth rate in the absence of density-dependent mortality, while the duration of these stages differ. Additional density-dependent mortality occurs at the larval stage, and we use a density-dependent equation of the form, $F(L)=\sqrt[{T}_{L}]{\alpha /(\alpha +L)}$, where *L* is the number of larvae, *T*_*L*_ is the duration of the larval stage, and *α* is a parameter determining the strength of density-dependence which is chosen to produce the desired equilibrium density of adult mosquitoes in the population. The form of the density-dependence equation is taken from Deredec *et al*.^4^, and may be thought of as a discrete version of the logistic growth model. It has the desirable feature that, while density-independent life parameters determine the population growth rate, a single parameter, *α*, determines the equilibrium density of adults (the value of this parameter may be determined from Equation S55, Supplementary Text S1). Adult males mate throughout their lifetime, while adult females mate only once, soon after that they emerge. Fecundity rates differ per genotype, with wild-type females laying *β* eggs per day, females heterozygous for the homing allele laying $\beta (1-s)$ eggs per day, HH females being infertile, and females of all other genotypes laying *β* eggs per day. Here, *s* represents the fractional reduction in fertility of females heterozygous for the homing allele. Initial estimates for these and other parameter values are provided in Supplementary Table 1. Equations describing this system are provided in Supplementary Text S1.

We use a stochastic implementation of this model to capture the random effects at low population sizes, for instance when the CRISPR-Cas9 system is causing significant population suppression. We assume that the number of eggs produced per day by females follows a Poisson distribution, the number of eggs having each genotype follows a multinomial distribution, and all survival/death events follow a Bernoulli distribution. Finally, female mate choice follows a binomial distribution with probabilities given by the relative frequency of each male genotype in the population.

### Construct Assembly

Gibson enzymatic assembly (EA) cloning method was used for all cloning^43^. To generate plasmid OA-16, components were cloned into the multiple cloning site (MCS) of a commonly used plasmid in the lab for *D*. *melanogaster* transformation that contains the *white* gene as a marker and an attB-docking site. Specifically, the *Drosophila* ubiquitin promoter^19^ was amplified from *D*. *melanogaster* genomic DNA using primers OA16-1 and OA16-2, and the SV40 3′UTR fragment was amplified from template pMos-3xP3-DsRed-attp (addgene plasmid #52904) using primers OA16-3 and OA16-4. The two RGRs were generated via sequential PCRs using primers OA16-5 and OA16-6 for the first PCR and OA16-5 and OA16-7 for the *white* RGR, and primers OA16-8 and OA16-5 for the first PCR and OA16-9 and OA16−10 for the *yellow* RGR. The construct was assembled in one step: the *D*. *melanogaster* attB stock plasmid was digested with AscI and XbaI, and the ubiquitin promoter, *white* RGR, *yellow* RGR, and the SV40 3′UTR were cloned in via EA cloning. A list of primer sequences used in the above construct assembly can be found in Supplementary Table 2.

### Fly Culture and Strains

Fly husbandry and crosses were performed under standard conditions at 25 °C. Rainbow Transgenics (Camarillo, CA) carried out all of the fly injections. The OA-16 construct was integrated into Bloomington Stock Center (BSC) fly strain 86Fa (BSC #24485: y^1^ M{vas-int.Dm}ZH-2A w^*^; M{3xP3-RFP.attP’}ZH-68E), and fly stock BSC#51324 (w[1118]; PBac{y[+mDint2] = vas-Cas9}VK00027) was used as the source of *vasa*-Cas9. For balancing chromosomes, fly stocks BSC#39631 (w[*]; wg[Sp-1]/CyO; P{ry[+t7.2] = neoFRT}82B lsn[SS6]/TM6C, Sb[1]) and BSC#2555 (CyO/sna[Sco]) were used. Homozygous stocks were first generated for 86Fa-OA-16 flies via use of balancer flies. Then, single homozygous female virgins and males were crossed out in triplicate to single male and female virgins, respectively, from the *vasa*-Cas9 line. The offspring (1765 in total) were scored for body color and eye color. The standard error of the mean (SEM) was calculated for each cross type and each phenotype using standard procedures.

### Sequencing to confirm mutations

Genomic DNA was extracted from single mutant flies using Qiagen DNeasy Blood and Tissue Kit (Hilden, Germany), and PCRs were set up using standard protocols to amplify regions of the *white* (primer set OA16-S1/OA16-S2) and *yellow* (primer set OA16-S3/OA16-S4) genes that span the cleavage site. Sequencing was performed by Source Bioscience (Nottingham, UK). Primer sequences can be found in Supplementary Table 2.

## Electronic supplementary material

Supplementary Info
