# Supplementary material for: Overcoming evolved resistance to population-suppressing homing-based gene drives
Source: Sci Rep. 2017 Jun 19;7:3776. doi: 10.1038/s41598-017-02744-7 (PMC5476637; doi:10.1038/s41598-017-02744-7)
Supplement: Supplementary file 1 — Supplementary Info [file 41598_2017_2744_MOESM1_ESM.pdf]

Supplementary Material

Title

Overcoming evolved resistance to population-suppressing homing-based gene drives

Authors

John M. Marshall[†§], Anna Buchman*, Héctor M. Sánchez C.[‡], Omar S. Akbari*[§]

Affiliations

[†]Divisions of Biostatistics and Epidemiology, School of Public Health, University of California, Berkeley, CA 94720, USA

*Department of Entomology, Center for Disease Vector Research, and Institute for Integrative Genome Biology, University of California, Riverside, CA 92521, USA

[‡]Bioinformatics Research Group, School of Medicine, Tecnológico de Monterrey, Estado de México, 52926, México

[§]**Corresponding Authors**

john.marshall@berkeley.edu, omar.akbari@ucr.edu

Keywords

Gene drive, Homing, Population suppression, Resistance alleles, Multiplexing, Mathematical model

Male

Female	HH	Hh	HR	hh	hR	RR
HH	1 HH	((1+e)/2) HH<br>((1-e-ρ)/2) Hh<br>(ρ/2) HR	(1/2) HH<br>(1/2) HR	(1) Hh	(1/2) Hh<br>(1/2) HR	(1) HR
Hh	((1+e)/2ρ) HH<br>((1-e-ρ)/2) Hh<br>(ρ/2) HR	Cross A	Cross B	((1+e)/2) Hh<br>((1-e-ρ)/2) hh<br>(ρ/2) hR	Cross D	((1+e)/2) HR<br>((1-e-ρ)/2) hR<br>(ρ/2) RR
HR	(1/2) HH<br>(1/2) HR	Cross C	(1/4) HH<br>(1/2) HR<br>(1/4) RR	(1/2) Hh<br>(1/2) hR	(1/4) Hh<br>(1/4) HR<br>(1/4) hR<br>(1/4) RR	(1/2) HR<br>(1/2) RR
hh	(1) Hh	((1+e)/2) Hh<br>((1-e-ρ)/2) hh<br>(ρ/2) HR	(1/2) Hh<br>(1/2) hR	(1) hh	(1/2) hR<br>(1/2) hh	(1) hR
hR	(1/2) Hh<br>(1/2) HR	Cross E	(1/4) Hh<br>(1/4) HR<br>(1/4) hR<br>(1/4) RR	(1/2) hR<br>(1/2) hh	(1/4) hh<br>(1/2) hR<br>(1/4) RR	(1/2) hR<br>(1/2) RR
RR	(1) HR	((1+e)/2) HR<br>((1-e-ρ)/2) hR<br>(ρ/2) RR	(1/2) HR<br>(1/2) RR	(1) hR	(1/2) hR<br>(1/2) RR	(1) RR

Cross A	Cross B/C	Cross D/E
$(((1+e)^2)/4)$ HH<br>(((1+e)(1-e-ρ))/2) Hh<br>(((1+e)ρ)/2) HR<br>$(((1-e-\rho)^2)/4)$ hh<br>(((1-e-ρ)ρ)/2) hR<br>$((\rho^2)/4)$ HR	((1+e)/4) HH<br>((1-e-ρ)/4) Hh<br>((1+e+ρ)/4) HR<br>((1-e-ρ)/4) hR<br>((ρ)/4) RR	((1+e)/4) Hh<br>((1+e)/4) HR<br>((1-e-ρ)/4) hh<br>((1-e)/4) hR<br>((ρ)/4) RR

Supplementary Figure 1. Crosses representing the inheritance pattern of an autosomal CRISPR-Cas9-based homing gene drive system. "H" denotes the CRISPR-Cas9-based homing construct, "h" denotes the corresponding wild-type allele, and "R" denotes a homing-resistant allele. Inheritance of the H allele is favored in heterozygous parents as determined by the homing rate, *e*. Homing-resistant alleles may be generated during the process of DNA cleavage and repair at a rate, *ρ*. Crosses involving HH females are shaded out as HH females are rendered infertile by the homing construct. The inheritance pattern of the homing and resistant alleles depicted here is incorporated into the population dynamic model described in the Materials and Methods.

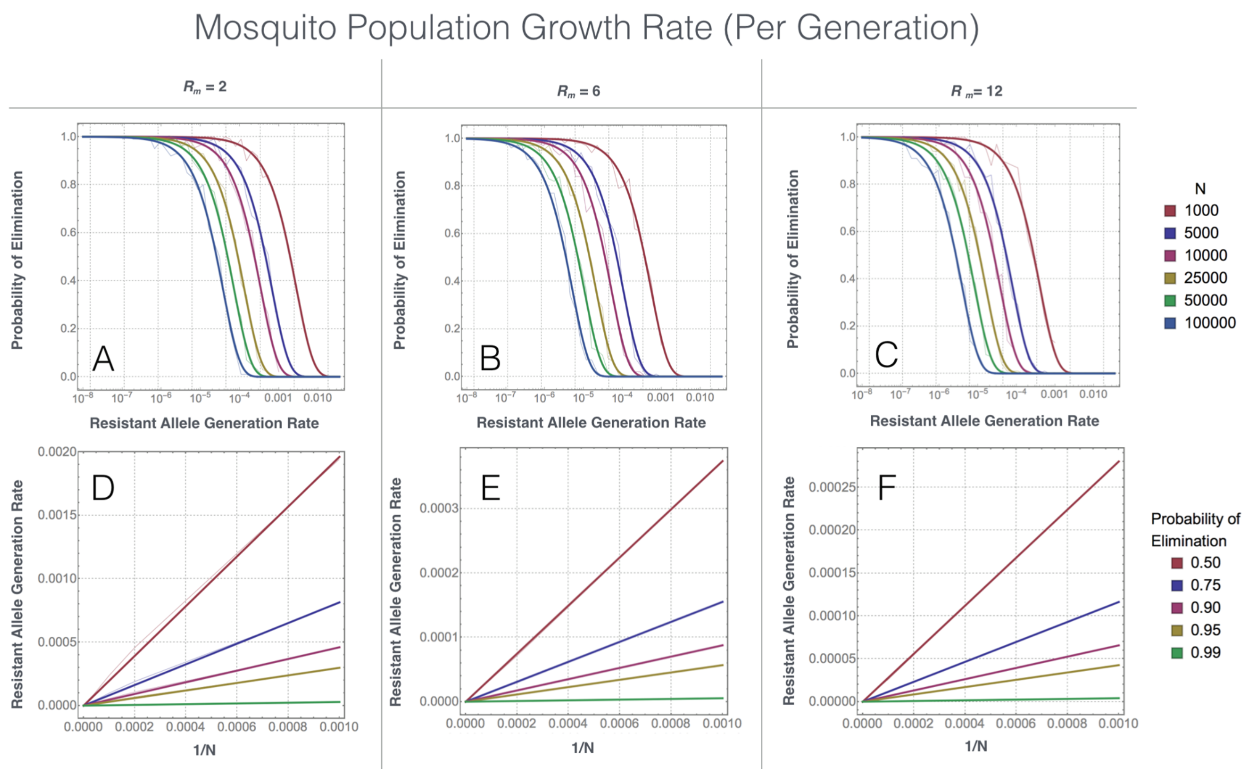

Supplementary Figure 2. Comparison of tolerable resistant allele generation rates for different mosquito population growth rates. (A-C) Elimination probability as a function of resistant allele generation rate for randomly mixing populations of size *N* and population growth rates of R_M = 2 (A), 6 (B) and 12 per generation (C). Sigmoidal curves are fitted to data points covering 30 resistant allele generation rates sampled logarithmically between 10^{-2} and 10^{-8}. (D-F) Linear relationship between 1/*N* and the resistant allele generation rate leading to a given probability of population elimination for population growth rates of R_M = 2 (D), 6 (E) and 12 per generation (F). Values of 1/*N* are as shown in panel A-C, and resistant allele generation rates are inferred from the sigmoid curves. Faint lines in all panels represent interpolation between simulated data points while solid lines represent fitted linear relationships. The linear relationship between 1/*N* and the resistant allele generation rate leading to a given elimination probability holds for low (2 per generation), medium (6 per generation) and high (12 per generation) growth rates; however the slopes vary.

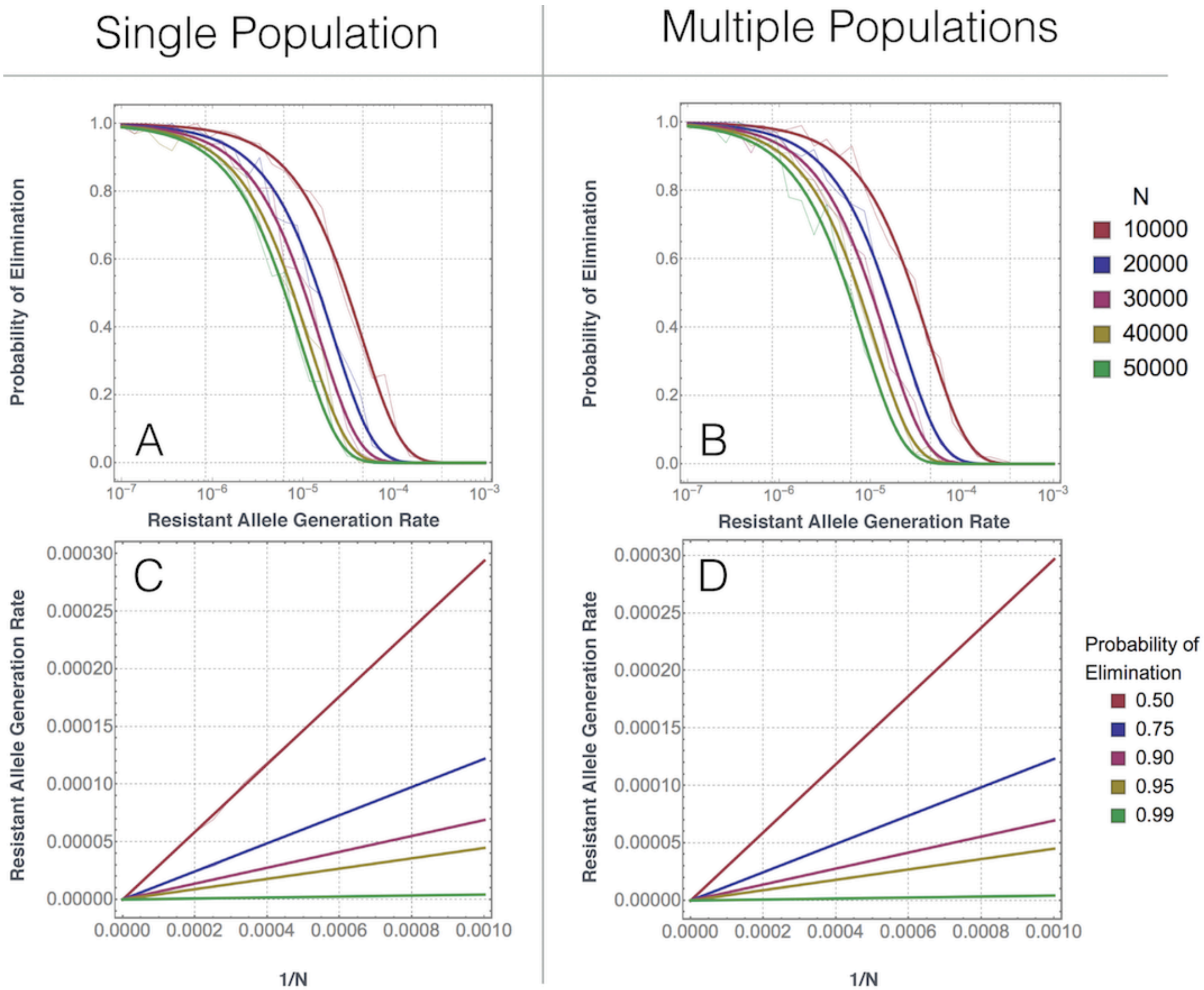

Supplementary Figure 3. Comparison of tolerable resistant allele generation rates with and without population structure. (A) Elimination probability as a function of resistant allele generation rate for randomly mixing populations of size, *N*, between 10,000 and 50,000. Sigmoidal curves are fitted to data points covering 30 resistant allele generation rates sampled logarithmically between 10^{-2} and 10^{-7}. (B) Linear relationship between 1/*N* and the resistant allele generation rate leading to a given probability of population elimination. Values of 1/*N* are as shown in panel A, and resistant allele generation rates are inferred from the sigmoid curves. (C) Elimination probability as a function of resistant allele generation rate for 1-5 randomly mixing populations, each of size 10,000, which exchange migrants with all other populations at a rate of 1% per adult mosquito per generation. (D) Linear relationship between 1/*N* and the resistant allele generation rate leading to a given probability of elimination, where *N* is the collective population size. Faint lines in all panels represent interpolation between simulated data points while solid lines represent fitted linear relationships. The linear relationship between

1/*N* and the resistant allele generation rate leading to a given elimination probability is unchanged by the presence of population structure, at least for the case explored here.

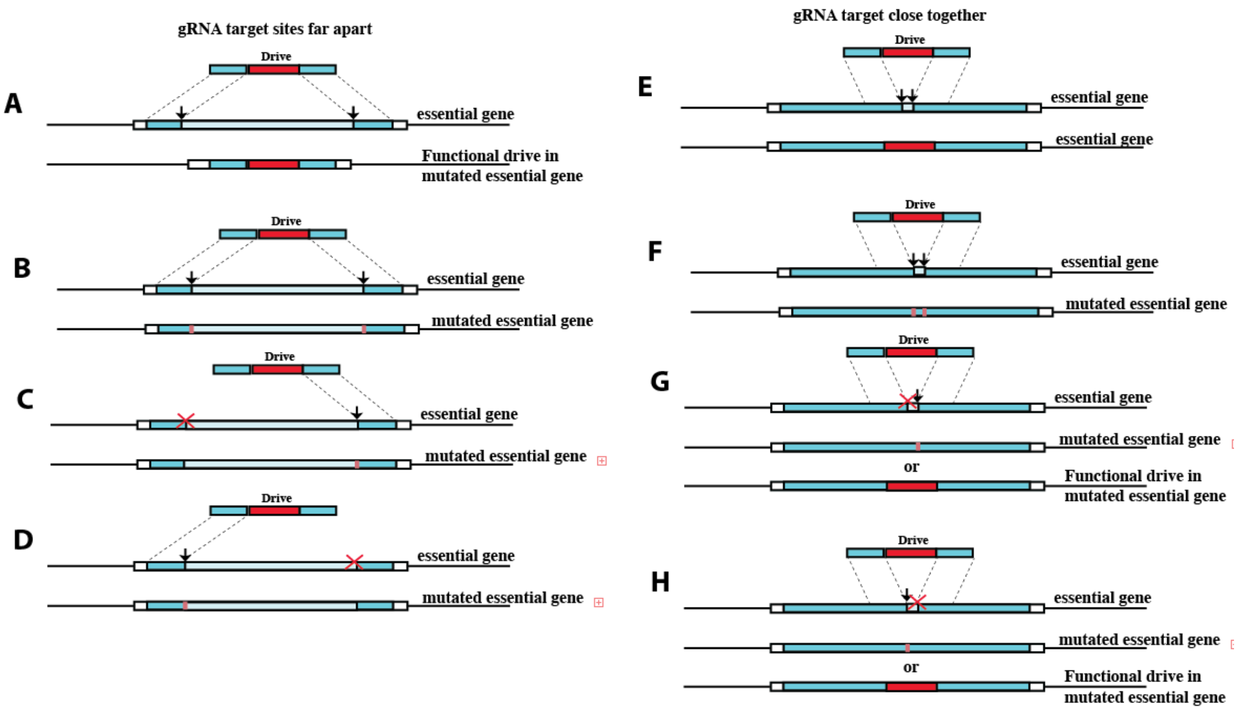

Supplementary Figure 4. Distance of multiplexed gRNA target sites may affect homing rates. We hypothesize that if a suppression based gene drive is designed to target an essential gene at two target sites that are far apart from each other, then four drive-induced possibilities may occur. These include, the drive being successfully copied over to the target allele via HDR following cleavage at both target sites (A), cleavage at both target sites and repair via NHEJ resulting in a mutated drive-resistant essential allele (B), or one of the target sites fails to get cleaved while the other gets cleaved and repaired via NHEJ generating a mutated essential gene (C,D). However, if the drive is designed to target an essential gene at two target sites that are relatively close to each other, but not too close to prevent Cas9 from generating mutations in adjacent target sites, then four different drive-induced possibilities may occur. These include, the drive being successfully copied over to the target allele via HDR following cleavage at both target sites (E), cleavage at both target sites and repair via NHEJ resulting in a mutated drive-resistant essential allele (F), or one of the target sites fails to get cleaved while the other gets cleaved and repaired via NHEJ generating either a mutated essential gene or a functional drive (G,H). Note – for all examples the mutated essential gene may or may not still be functional, however it should be resistant to the endonuclease in the drive due to the mutated gRNA target site.

Supplementary Text S1:

Model equations for CRISPR-Cas9-based population dynamics in *Anopheles gambiae*:
In the main text we describe a stochastic framework for modeling the spread of a CRISPR-Cas9-based gene drive system targeting a female fertility gene through a randomly mating population; however equations were left out for brevity. These are included here for completeness.

Using *An. gambiae* as a case study, we adapt the modeling framework of Deredec *et al.* (2011) to describe the spread of the CRISPR and homing-resistant alleles through a discrete, density-dependent population with time steps of one day. In this model, the mosquito life cycle is divided into four life stages – egg, larva, pupa and adult (both male and female) – denoted by the subscripts "E", "L", "P" and "M", respectively. The daily, density-independent mortality rates for the juvenile stages are assumed to be identical ($\mu_E = \mu_L = \mu_P$) and are chosen for consistency with the population growth rate in the absence of density-dependent mortality, R_M. The duration of these stages differ and are given by T_E, T_L and T_P. The probability of surviving any of the juvenile stages in a density-independent setting is given by $\theta_i = (1-\mu_i)^{T_i}$, where $i \in \{E, L, P\}$; however additional density-dependent mortality, $1 - F(L)$, occurs at the larval stage. We use a density-dependent equation of the form, $F(L) = \sqrt[T_L]{\alpha / (\alpha + L)}$, where α is a parameter influencing the strength of density-dependence and is chosen to produce the desired equilibrium density of adult mosquitoes in the population. For adult mosquitoes, the mortality rate is denoted by μ_M. Fecundity rates are allowed to differ, with wild-type females laying $\beta_{hh} = \beta$ eggs per day, heterozygous females (Hh and HR) laying $\beta_{Hh} = \beta_{HR} = \beta(1-s)$ eggs per day, HH females being infertile ($\beta_{HH} = 0$), and females of other genotypes (hR and RR) laying $\beta_{hR} = \beta_{RR} = \beta$ eggs per day. Here, *s* represents the fractional reduction in fertility of females heterozygous for the homing allele. Initial estimates for these and other parameter values are provided in Supplementary Table 1.

With this framework in place, the dynamics of the population can be described by equations for the number of larvae and adults belonging to each genotype at time *t*. The number of larvae is needed to determine the strength of density-dependence. Since HH female infertility is irrelevant

at the larval stage, we describe the total larval population size at time *t* as,

$$
\begin{aligned}
L_t &= L_{t-1}(1-\mu_L)F(L_{t-1}) + \sum_{\substack{x,y\in\{HH,Hh,\\HR,hh,hR,RR\}}} \left(\beta_x M^{x,y}_{t-T_E}\theta_E\right) \\
&- \sum_{\substack{x,y\in\{HH,Hh,\\HR,hh,hR,RR\}}} \left(\beta_x M^{x,y}_{t-T_E-T_L}\theta_E\theta_L \prod_{i=1}^{T_L} F(L_{t-i})\right)
\end{aligned} \qquad \text{(S1)}
$$

Here, the first term accounts for survival of larvae (denoted at time *t* by L_t) from one day to the next, the second term accounts for newly hatching eggs of any genotype from females of any genotype *x* that have mated with males of any genotype *y* (denoted at time *t* by $M_t^{x,y}$), and the third term accounts for transformation of larvae into pupae for juvenile stages resulting from the same crosses.

Adult males and females are treated slightly differently in this framework since it is assumed that female mosquitoes only mate once, while male mosquitoes may mate throughout their lifetime. For example, the number of male adults of genotype HH at time *t* is given by,

$$
\begin{aligned}
M_t^{HH} &= M_{t-1}^{HH}(1-\mu_M) \\
&+ \frac{1}{2}\begin{pmatrix} E^{HH}_{Hh,HH} + E^{HH}_{Hh,Hh} + E^{HH}_{Hh,HR} \\ +E^{HH}_{HR,HH} + E^{HH}_{HR,Hh} + E^{HH}_{HR,HR} \end{pmatrix} \theta_E\theta_L \prod_{i=1}^{T_L} F(L_{t-i-T_P})\theta_P(1-\mu_M)
\end{aligned} \qquad \text{(S2)}
$$

Here, the first term accounts for survival of HH adult males (denoted at time *t* by M_t^{HH}) from one day to the next, and the second term accounts for transformation of HH pupae into adult males, where these pupae result from crosses between Hh and HR females with HH, Hh and HR males. The number of eggs of genotype *x* produced by adult females of genotype *y* that have mated with a male of genotype *z* is given by $E^x_{y,z}$. These quantities are time-dependent and the product of the fecundity of the female genotype, β_y, the number of females having the given mated genotype, $M^{y,z}$, and the proportion of offspring of this mated genotype having the genotype *z* (depicted in the crosses shown in Supplementary Figure 1). The numbers of HH eggs from each cross in Equation S2 are given by the following equations:

$$E_{Hh,HH}^{HH} = \beta(1-s)M_{t-T_E-T_L-T_P}^{Hh,HH}\left(\frac{1+e}{2}\right) , \tag{S3}$$

$$E_{Hh,Hh}^{HH} = \beta(1-s)M_{t-T_E-T_L-T_P}^{Hh,Hh}\left(\frac{(1+e)^2}{4}\right) , \tag{S4}$$

$$E_{Hh,HR}^{HH} = \beta(1-s)M_{t-T_E-T_L-T_P}^{Hh,HR}\left(\frac{(1+e)}{4}\right) , \tag{S5}$$

$$E_{HR,HH}^{HH} = \beta(1-s)M_{t-T_E-T_L-T_P}^{HR,HH}\left(\frac{1}{2}\right) , \tag{S6}$$

$$E_{HR,Hh}^{HH} = \beta(1-s)M_{t-T_E-T_L-T_P}^{HR,Hh}\left(\frac{(1+e)}{4}\right) , \tag{S7}$$

$$E_{HR,HR}^{HH} = \beta(1-s)M_{t-T_E-T_L-T_P}^{HR,HR}\left(\frac{1}{4}\right) . \tag{S8}$$

Crosses involving HH females are not included here, as these females are rendered infertile by the CRISPR construct.

Females, on the other hand, are assumed to mate only once and on the same day that they emerge. They can therefore be described by both their genotype and the genotype of the male with whom they mated. For example, the number of female adults at time *t* of genotype HH that have mated with hh males is given by,

$$\begin{aligned} M_t^{HH,hh} &= M_{t-1}^{HH,hh}(1-\mu_M) \\ &+\frac{1}{2}\begin{pmatrix} E_{Hh,HH}^{HH} + E_{Hh,Hh}^{HH} + E_{Hh,HR}^{HH} \\ +E_{HR,HH}^{HH} + E_{HR,Hh}^{HH} + E_{HR,HR}^{HH} \end{pmatrix}\theta_E\theta_L\prod_{i=1}^{T_L}F(L_{t-i-T_P})\theta_P(1-\mu_M) \\ &\times\frac{M_{t-1}^{hh}}{M_{t-1}^{HH}+M_{t-1}^{Hh}+M_{t-1}^{HR}+M_{t-1}^{hh}+M_{t-1}^{hR}+M_{t-1}^{RR}} \end{aligned} . \tag{S9}$$

Here, the first term accounts for survival of HH adult females that have mated with hh males (denoted at time *t* by $M_t^{HH,hh}$) from one day to the next, and the second term accounts for transformation of HH pupae into adult females, where these pupae result from crosses between Hh and HR females with HH, Hh and HR males. This term is multiplied by the fraction of the adult male population having the genotype hh. Equations for all other adult genotypes are treated analogously as follows.

Equation S2 describes the number of adult males of genotype HH over time. There are five other male genotypes – Hh, HR, hh, hR and RR – denoted by the variables M_t^{HH}, M_t^{Hh}, M_t^{HR}, M_t^{hh}, M_t^{hR} and M_t^{RR} respectively, and described by the following equations:

$$M_t^{Hh} = M_{t-1}^{Hh}(1-\mu_M) + \frac{1}{2}\begin{pmatrix} E_{Hh,HH}^{Hh} + E_{Hh,Hh}^{Hh} + E_{Hh,HR}^{Hh} + E_{Hh,hh}^{Hh} \\ + E_{Hh,hR}^{Hh} + E_{HR,Hh}^{Hh} + E_{HR,hh}^{Hh} + E_{HR,hR}^{Hh} \\ + E_{hh,HH}^{Hh} + E_{hh,Hh}^{Hh} + E_{hh,HR}^{Hh} + E_{hR,HH}^{Hh} \\ + E_{hR,Hh}^{Hh} + E_{hR,HR}^{Hh} \end{pmatrix} \theta_E \theta_L \prod_{i=1}^{T_L} F(L_{t-i-T_P}) \theta_P (1-\mu_M), \tag{S10}$$

$$M_t^{HR} = M_{t-1}^{HR}(1-\mu_M) + \frac{1}{2}\begin{pmatrix} E_{Hh,HH}^{HR} + E_{Hh,Hh}^{HR} + E_{Hh,HR}^{HR} + E_{Hh,hR}^{HR} \\ + E_{Hh,RR}^{HR} + E_{HR,HH}^{HR} + E_{HR,Hh}^{HR} + E_{HR,HR}^{HR} \\ + E_{HR,hR}^{HR} + E_{HR,RR}^{HR} + E_{hR,HH}^{HR} + E_{hR,Hh}^{HR} \\ + E_{hR,HR}^{HR} + E_{RR,HH}^{HR} + E_{RR,Hh}^{HR} + E_{RR,HR}^{HR} \end{pmatrix} \theta_E \theta_L \prod_{i=1}^{T_L} F(L_{t-i-T_P}) \theta_P (1-\mu_M), \tag{S11}$$

$$M_t^{hh} = M_{t-1}^{hh}(1-\mu_M) + \frac{1}{2}\begin{pmatrix} E_{Hh,Hh}^{hh} + E_{Hh,hh}^{hh} + E_{Hh,hR}^{hh} + E_{hh,Hh}^{hh} + E_{hh,hh}^{hh} \\ + E_{hh,hR}^{hh} + E_{hR,Hh}^{hh} + E_{hR,hh}^{hh} + E_{hR,hR}^{hh} \end{pmatrix} \theta_E \theta_L \prod_{i=1}^{T_L} F(L_{t-i-T_P}) \theta_P (1-\mu_M), \tag{S12}$$

$$M_t^{hR} = M_{t-1}^{hR}(1-\mu_M) + \frac{1}{2}\begin{pmatrix} E_{Hh,Hh}^{hR} + E_{Hh,HR}^{hR} + E_{Hh,hh}^{hR} + E_{Hh,hR}^{hR} + E_{Hh,RR}^{hR} \\ + E_{HR,Hh}^{hR} + E_{HR,hh}^{hR} + E_{HR,hR}^{hR} + E_{hh,Hh}^{hR} + E_{hh,HR}^{hR} \\ + E_{hh,hR}^{hR} + E_{hh,RR}^{hR} + E_{hR,Hh}^{hR} + E_{hR,HR}^{hR} + E_{hR,hh}^{hR} \\ + E_{hR,hR}^{hR} + E_{hR,RR}^{hR} + E_{RR,Hh}^{hR} + E_{RR,hh}^{hR} + E_{RR,hR}^{hR} \end{pmatrix} \theta_E \theta_L \prod_{i=1}^{T_L} F(L_{t-i-T_P}) \theta_P (1-\mu_M), \tag{S13}$$

$$M_t^{RR} = M_{t-1}^{RR}(1-\mu_M) + \frac{1}{2}\begin{pmatrix} E_{Hh,Hh}^{RR} + E_{Hh,HR}^{RR} + E_{Hh,hR}^{RR} + E_{Hh,RR}^{RR} + E_{HR,Hh}^{RR} \\ + E_{HR,HR}^{RR} + E_{HR,hR}^{RR} + E_{HR,RR}^{RR} + E_{hR,Hh}^{RR} + E_{hR,HR}^{RR} \\ + E_{hR,hR}^{RR} + E_{hR,RR}^{RR} + E_{RR,Hh}^{RR} + E_{RR,HR}^{RR} + E_{RR,hR}^{RR} \\ + E_{RR,RR}^{RR} \end{pmatrix} \theta_E \theta_L \prod_{i=1}^{T_L} F(L_{t-i-T_P}) \theta_P (1-\mu_M). \tag{S14}$$

Here, the first term accounts for adult survival for each genotype from one day to the next, and the second term accounts for transformation of pupae into adult males for each genotype, where pupae result from the crosses depicted in Supplementary Figure 1. The number of eggs of genotype *x* produced by adult females of genotype *y* that have mated with a male of genotype *z* is given by $E^{x}_{y,z}$. These quantities are the product of the fecundity of the female genotype, β_y, the number of females having the given mated genotype, $M^{y,z}$, and the proportion of offspring of this mated genotype having the genotype *z*. The numbers of eggs of all genotypes for each of the crosses depicted in Supplementary Figure 1 are given by the following equations:

Eggs produced by *Hh* females:

$$(E^{HH}_{Hh,HH}, E^{Hh}_{Hh,HH}, E^{HR}_{Hh,HH}) = \beta(1-s)M^{Hh,HH}_{t-T_E-T_L-T_P}\left(\frac{1+e}{2}, \frac{1-e-\rho}{2}, \frac{\rho}{2}\right), \quad \text{(S15)}$$

$$\begin{pmatrix} E^{HH}_{Hh,Hh}, E^{Hh}_{Hh,Hh}, E^{HR}_{Hh,Hh}, \\ E^{hh}_{Hh,Hh}, E^{hR}_{Hh,Hh}, E^{RR}_{Hh,Hh} \end{pmatrix} = \beta(1-s)M^{Hh,Hh}_{t-T_E-T_L-T_P}\begin{pmatrix} \frac{(1+e)^2}{4}, \frac{(1+e)(1-e-\rho)}{2}, \frac{(1+e)\rho}{2}, \\ \frac{(1-e-\rho)^2}{4}, \frac{(1-e-\rho)\rho}{2}, \frac{\rho^2}{4} \end{pmatrix}, \quad \text{(S16)}$$

$$\begin{pmatrix} E^{HH}_{Hh,HR}, E^{Hh}_{Hh,HR}, E^{HR}_{Hh,HR}, \\ E^{hR}_{Hh,HR}, E^{RR}_{Hh,HR} \end{pmatrix} = \beta(1-s)M^{Hh,HR}_{t-T_E-T_L-T_P}\begin{pmatrix} \frac{(1+e)}{4}, \frac{(1-e-\rho)}{4}, \frac{(1+e)+\rho}{4}, \\ \frac{(1-e-\rho)}{4}, \frac{\rho}{4} \end{pmatrix}, \quad \text{(S17)}$$

$$(E^{Hh}_{Hh,hh}, E^{hh}_{Hh,hh}, E^{hR}_{Hh,hh}) = \beta(1-s)M^{Hh,hh}_{t-T_E-T_L-T_P}\left(\frac{1+e}{2}, \frac{1-e-\rho}{2}, \frac{\rho}{2}\right), \quad \text{(S18)}$$

$$\begin{pmatrix} E^{Hh}_{Hh,hR}, E^{HR}_{Hh,hR}, E^{hh}_{Hh,hR}, \\ E^{hR}_{Hh,hR}, E^{RR}_{Hh,hR} \end{pmatrix} = \beta(1-s)M^{Hh,hR}_{t-T_E-T_L-T_P}\begin{pmatrix} \frac{(1+e)}{4}, \frac{(1+e)}{4}, \frac{(1-e-\rho)}{4}, \\ \frac{(1-e-\rho)+\rho}{4}, \frac{\rho}{4} \end{pmatrix}, \quad \text{(S19)}$$

$$(E^{HR}_{Hh,RR}, E^{hR}_{Hh,RR}, E^{RR}_{Hh,RR}) = \beta(1-s)M^{Hh,RR}_{t-T_E-T_L-T_P}\left(\frac{1+e}{2}, \frac{1-e-\rho}{2}, \frac{\rho}{2}\right). \quad \text{(S20)}$$

Eggs produced by *HR* females:

$$(E^{HH}_{HR,HH}, E^{HR}_{HR,HH}) = \beta(1-s)M^{HR,HH}_{t-T_E-T_L-T_P}\left(\frac{1}{2}, \frac{1}{2}\right), \quad \text{(S21)}$$

$$\begin{pmatrix} E^{HH}_{HR,Hh}, E^{Hh}_{HR,Hh}, E^{HR}_{HR,Hh}, \\ E^{hR}_{HR,Hh}, E^{RR}_{HR,Hh} \end{pmatrix} = \beta(1-s)M^{HR,Hh}_{t-T_E-T_L-T_P} \begin{pmatrix} \frac{(1+e)}{4}, \frac{(1-e-\rho)}{4}, \frac{(1+e)+\rho}{4}, \\ \frac{(1-e-\rho)}{4}, \frac{\rho}{4} \end{pmatrix}, \tag{S22}$$

$$(E^{HH}_{HR,HR}, E^{HR}_{HR,HR}, E^{RR}_{HR,HR}) = \beta(1-s)M^{HR,HR}_{t-T_E-T_L-T_P}\left(\frac{1}{4}, \frac{1}{2}, \frac{1}{4}\right), \tag{S23}$$

$$(E^{Hh}_{HR,hh}, E^{hR}_{HR,hh}) = \beta(1-s)M^{HR,hh}_{t-T_E-T_L-T_P}\left(\frac{1}{2}, \frac{1}{2}\right), \tag{S24}$$

$$(E^{Hh}_{HR,hR}, E^{HR}_{HR,hR}, E^{hR}_{HR,hR}, E^{RR}_{HR,hR}) = \beta(1-s)M^{HR,hR}_{t-T_E-T_L-T_P}\left(\frac{1}{4}, \frac{1}{4}, \frac{1}{4}, \frac{1}{4}\right), \tag{S25}$$

$$(E^{HR}_{HR,RR}, E^{RR}_{HR,RR}) = \beta(1-s)M^{HR,RR}_{t-T_E-T_L-T_P}\left(\frac{1}{2}, \frac{1}{2}\right). \tag{S26}$$

Eggs produced by *hh* females:

$$E^{Hh}_{hh,HH} = \beta M^{hh,HH}_{t-T_E-T_L-T_P}, \tag{S27}$$

$$(E^{Hh}_{hh,Hh}, E^{hh}_{hh,Hh}, E^{hR}_{hh,Hh}) = \beta M^{hh,Hh}_{t-T_E-T_L-T_P}\left(\frac{1+e}{2}, \frac{1-e-\rho}{2}, \frac{\rho}{2}\right), \tag{S28}$$

$$(E^{Hh}_{hh,HR}, E^{hR}_{hh,HR}) = \beta M^{hh,HR}_{t-T_E-T_L-T_P}\left(\frac{1}{2}, \frac{1}{2}\right), \tag{S29}$$

$$E^{hh}_{hh,hh} = \beta M^{hh,hh}_{t-T_E-T_L-T_P}, \tag{S30}$$

$$(E^{hh}_{hh,hR}, E^{hR}_{hh,hR}) = \beta M^{hh,hR}_{t-T_E-T_L-T_P}\left(\frac{1}{2}, \frac{1}{2}\right), \tag{S31}$$

$$E^{hR}_{hh,RR} = \beta M^{hh,RR}_{t-T_E-T_L-T_P}. \tag{S32}$$

Eggs produced by *hR* females:

$$(E^{Hh}_{hR,HH}, E^{HR}_{hR,HH}) = \beta M^{hR,HH}_{t-T_E-T_L-T_P}\left(\frac{1}{2}, \frac{1}{2}\right), \tag{S33}$$

$$\begin{pmatrix} E^{Hh}_{hR,Hh}, E^{HR}_{hR,Hh}, E^{hh}_{hR,Hh}, \\ E^{hR}_{hR,Hh}, E^{RR}_{hR,Hh} \end{pmatrix} = \beta M^{hR,Hh}_{t-T_E-T_L-T_P} \begin{pmatrix} \frac{(1+e)}{4}, \frac{(1+e)}{4}, \frac{(1-e-\rho)}{4}, \\ \frac{(1-e-\rho)+\rho}{4}, \frac{\rho}{4} \end{pmatrix}, \tag{S34}$$

$$(E^{Hh}_{hR,HR}, E^{HR}_{hR,HR}, E^{hR}_{hR,HR}, E^{RR}_{hR,HR}) = \beta M^{hR,HR}_{t-T_E-T_L-T_P}\left(\frac{1}{4}, \frac{1}{4}, \frac{1}{4}, \frac{1}{4}\right), \tag{S35}$$

$$(E_{hR,hh}^{hh}, E_{hR,hh}^{hR}) = \beta M_{t-T_E-T_L-T_P}^{hR,hh} \left(\frac{1}{2}, \frac{1}{2} \right) \ , \tag{S36}$$

$$(E_{hR,hR}^{hh}, E_{hR,hR}^{hR}, E_{hR,hR}^{RR}) = \beta M_{t-T_E-T_L-T_P}^{hR,hR} \left(\frac{1}{4}, \frac{1}{2}, \frac{1}{4} \right) \ , \tag{S37}$$

$$(E_{hR,RR}^{hR}, E_{hR,RR}^{RR}) = \beta M_{t-T_E-T_L-T_P}^{hR,RR} \left(\frac{1}{2}, \frac{1}{2} \right) \ . \tag{S38}$$

Eggs produced by *RR* females:

$$E_{RR,HH}^{HR} = \beta M_{t-T_E-T_L-T_P}^{RR,HH} \ , \tag{S39}$$

$$(E_{RR,Hh}^{HR}, E_{RR,Hh}^{hR}, E_{RR,Hh}^{RR}) = \beta M_{t-T_E-T_L-T_P}^{RR,Hh} \left(\frac{1+e}{2}, \frac{1-e-\rho}{2}, \frac{\rho}{2} \right) \ , \tag{S40}$$

$$(E_{RR,HR}^{HR}, E_{RR,HR}^{RR}) = \beta M_{t-T_E-T_L-T_P}^{RR,HR} \left(\frac{1}{2}, \frac{1}{2} \right) \ , \tag{S41}$$

$$E_{RR,hh}^{hR} = \beta M_{t-T_E-T_L-T_P}^{RR,hh} \ , \tag{S42}$$

$$(E_{RR,hR}^{hR}, E_{RR,hR}^{RR}) = \beta M_{t-T_E-T_L-T_P}^{RR,hR} \left(\frac{1}{2}, \frac{1}{2} \right) \ , \tag{S43}$$

$$E_{RR,RR}^{RR} = \beta M_{t-T_E-T_L-T_P}^{RR,RR} \ . \tag{S44}$$

As mentioned earlier, crosses involving HH females are not included here, as these females are rendered infertile by the CRISPR construct.

Females are assumed to mate only once and on the same day that they emerge so can therefore be described by both their genotype and the genotype of the male with whom they mated. Equation S9 describes the number of female adults of genotype HH that have mated with hh males over time. The other mated female genotypes are described by the following equations:

$$
\begin{aligned}
&(M_t^{HH,HH}, M_t^{HH,Hh}, M_t^{HH,HR}, M_t^{HH,hh}, M_t^{HH,hR}, M_t^{HH,RR}) \\
&= (M_{t-1}^{HH,HH}, M_{t-1}^{HH,Hh}, M_{t-1}^{HH,HR}, M_{t-1}^{HH,hh}, M_{t-1}^{HH,hR}, M_{t-1}^{HH,RR})(1-\mu_M) \\
&+\frac{1}{2}\begin{pmatrix} E_{Hh,HH}^{HH} + E_{Hh,Hh}^{HH} + E_{Hh,HR}^{HH} \\ +E_{HR,HH}^{HH} + E_{HR,Hh}^{HH} + E_{HR,HR}^{HH} \end{pmatrix} \theta_E \theta_L \prod_{i=1}^{T_L} F(L_{t-i-T_P}) \theta_P (1-\mu_M) \ , \\
&\times \frac{(M_{t-1}^{HH}, M_{t-1}^{Hh}, M_{t-1}^{HR}, M_{t-1}^{hh}, M_{t-1}^{hR}, M_{t-1}^{RR})}{M_{t-1}^{HH} + M_{t-1}^{Hh} + M_{t-1}^{HR} + M_{t-1}^{hh} + M_{t-1}^{hR} + M_{t-1}^{RR}}
\end{aligned}
\tag{S45}
$$

$$
\begin{aligned}
&(M_t^{Hh,HH}, M_t^{Hh,Hh}, M_t^{Hh,HR}, M_t^{Hh,hh}, M_t^{Hh,hR}, M_t^{Hh,RR}) \\
&= (M_{t-1}^{Hh,HH}, M_{t-1}^{Hh,Hh}, M_{t-1}^{Hh,HR}, M_{t-1}^{Hh,hh}, M_{t-1}^{Hh,hR}, M_{t-1}^{Hh,RR})(1-\mu_M) \\
&+\frac{1}{2}\begin{pmatrix} E_{Hh,HH}^{Hh} + E_{Hh,Hh}^{Hh} + E_{Hh,HR}^{Hh} + E_{Hh,hh}^{Hh} \\ +E_{Hh,hR}^{Hh} + E_{HR,Hh}^{Hh} + E_{HR,hh}^{Hh} + E_{HR,hR}^{Hh} \\ +E_{hh,HH}^{Hh} + E_{hh,Hh}^{Hh} + E_{hh,HR}^{Hh} + E_{hR,HH}^{Hh} \\ +E_{hR,Hh}^{Hh} + E_{hR,HR}^{Hh} \end{pmatrix} \theta_E \theta_L \prod_{i=1}^{T_L} F(L_{t-i-T_P}) \theta_P (1-\mu_M) \ , \\
&\times \frac{(M_{t-1}^{HH}, M_{t-1}^{Hh}, M_{t-1}^{HR}, M_{t-1}^{hh}, M_{t-1}^{hR}, M_{t-1}^{RR})}{M_{t-1}^{HH} + M_{t-1}^{Hh} + M_{t-1}^{HR} + M_{t-1}^{hh} + M_{t-1}^{hR} + M_{t-1}^{RR}}
\end{aligned}
\tag{S46}
$$

$$
\begin{aligned}
&(M_t^{HR,HH}, M_t^{HR,Hh}, M_t^{HR,HR}, M_t^{HR,hh}, M_t^{HR,hR}, M_t^{HR,RR}) \\
&= (M_{t-1}^{HR,HH}, M_{t-1}^{HR,Hh}, M_{t-1}^{HR,HR}, M_{t-1}^{HR,hh}, M_{t-1}^{HR,hR}, M_{t-1}^{HR,RR})(1-\mu_M) \\
&+\frac{1}{2}\begin{pmatrix} E_{Hh,HH}^{HR} + E_{Hh,Hh}^{HR} + E_{Hh,HR}^{HR} + E_{Hh,hR}^{HR} \\ +E_{Hh,RR}^{HR} + E_{HR,HH}^{HR} + E_{HR,Hh}^{HR} + E_{HR,HR}^{HR} \\ +E_{HR,hR}^{HR} + E_{HR,RR}^{HR} + E_{hR,HH}^{HR} + E_{hR,Hh}^{HR} \\ +E_{hR,HR}^{HR} + E_{RR,HH}^{HR} + E_{RR,Hh}^{HR} + E_{RR,HR}^{HR} \end{pmatrix} \theta_E \theta_L \prod_{i=1}^{T_L} F(L_{t-i-T_P}) \theta_P (1-\mu_M) \ , \\
&\times \frac{(M_{t-1}^{HH}, M_{t-1}^{Hh}, M_{t-1}^{HR}, M_{t-1}^{hh}, M_{t-1}^{hR}, M_{t-1}^{RR})}{M_{t-1}^{HH} + M_{t-1}^{Hh} + M_{t-1}^{HR} + M_{t-1}^{hh} + M_{t-1}^{hR} + M_{t-1}^{RR}}
\end{aligned}
\tag{S47}
$$

$$
\begin{aligned}
&(M_t^{hh,HH}, M_t^{hh,Hh}, M_t^{hh,HR}, M_t^{hh,hh}, M_t^{hh,hR}, M_t^{hh,RR}) \\
&= (M_{t-1}^{hh,HH}, M_{t-1}^{hh,Hh}, M_{t-1}^{hh,HR}, M_{t-1}^{hh,hh}, M_{t-1}^{hh,hR}, M_{t-1}^{hh,RR})(1-\mu_M) \\
&+\frac{1}{2}\begin{pmatrix} E_{Hh,Hh}^{hh} + E_{Hh,hh}^{hh} + E_{Hh,hR}^{hh} + E_{hh,Hh}^{hh} + E_{hh,hh}^{hh} \\ +E_{hh,hR}^{hh} + E_{hR,Hh}^{hh} + E_{hR,hh}^{hh} + E_{hR,hR}^{hh} \end{pmatrix} \theta_E \theta_L \prod_{i=1}^{T_L} F(L_{t-i-T_P}) \theta_P (1-\mu_M) \ , \\
&\times \frac{(M_{t-1}^{HH}, M_{t-1}^{Hh}, M_{t-1}^{HR}, M_{t-1}^{hh}, M_{t-1}^{hR}, M_{t-1}^{RR})}{M_{t-1}^{HH} + M_{t-1}^{Hh} + M_{t-1}^{HR} + M_{t-1}^{hh} + M_{t-1}^{hR} + M_{t-1}^{RR}}
\end{aligned}
\tag{S48}
$$

$$(M_t^{hR,HH}, M_t^{hR,Hh}, M_t^{hR,HR}, M_t^{hR,hh}, M_t^{hR,hR}, M_t^{hR,RR})$$
$$= (M_{t-1}^{hR,HH}, M_{t-1}^{hR,Hh}, M_{t-1}^{hR,HR}, M_{t-1}^{hR,hh}, M_{t-1}^{hR,hR}, M_{t-1}^{hR,RR})(1-\mu_M)$$
$$+\frac{1}{2}\begin{pmatrix} E_{Hh,Hh}^{hR} + E_{Hh,HR}^{hR} + E_{Hh,hh}^{hR} + E_{Hh,hR}^{hR} + E_{Hh,RR}^{hR} \\ +E_{HR,Hh}^{hR} + E_{HR,hh}^{hR} + E_{HR,hR}^{hR} + E_{hh,Hh}^{hR} + E_{hh,HR}^{hR} \\ +E_{hh,hR}^{hR} + E_{hh,RR}^{hR} + E_{hR,Hh}^{hR} + E_{hR,HR}^{hR} + E_{hR,hh}^{hR} \\ +E_{hR,hR}^{hR} + E_{hR,RR}^{hR} + E_{RR,Hh}^{hR} + E_{RR,hh}^{hR} + E_{RR,hR}^{hR} \end{pmatrix} \theta_E \theta_L \prod_{i=1}^{T_L} F(L_{t-i-T_P}) \theta_P (1-\mu_M) \ , \tag{S49}$$
$$\times \frac{(M_{t-1}^{HH}, M_{t-1}^{Hh}, M_{t-1}^{HR}, M_{t-1}^{hh}, M_{t-1}^{hR}, M_{t-1}^{RR})}{M_{t-1}^{HH} + M_{t-1}^{Hh} + M_{t-1}^{HR} + M_{t-1}^{hh} + M_{t-1}^{hR} + M_{t-1}^{RR}}$$

$$(M_t^{RR,HH}, M_t^{RR,Hh}, M_t^{RR,HR}, M_t^{RR,hh}, M_t^{RR,hR}, M_t^{RR,RR})$$
$$= (M_{t-1}^{RR,HH}, M_{t-1}^{RR,Hh}, M_{t-1}^{RR,HR}, M_{t-1}^{RR,hh}, M_{t-1}^{RR,hR}, M_{t-1}^{RR,RR})(1-\mu_M)$$
$$+\frac{1}{2}\begin{pmatrix} E_{Hh,Hh}^{RR} + E_{Hh,HR}^{RR} + E_{Hh,hR}^{RR} + E_{Hh,RR}^{RR} + E_{HR,Hh}^{RR} \\ +E_{HR,HR}^{RR} + E_{HR,hR}^{RR} + E_{HR,RR}^{RR} + E_{hR,Hh}^{RR} + E_{hR,HR}^{RR} \\ +E_{hR,hR}^{RR} + E_{hR,RR}^{RR} + E_{RR,Hh}^{RR} + E_{RR,HR}^{RR} + E_{RR,hR}^{RR} \\ +E_{RR,RR}^{RR} \end{pmatrix} \theta_E \theta_L \prod_{i=1}^{T_L} F(L_{t-i-T_P}) \theta_P (1-\mu_M) \ . \tag{S50}$$
$$\times \frac{(M_{t-1}^{HH}, M_{t-1}^{Hh}, M_{t-1}^{HR}, M_{t-1}^{hh}, M_{t-1}^{hR}, M_{t-1}^{RR})}{M_{t-1}^{HH} + M_{t-1}^{Hh} + M_{t-1}^{HR} + M_{t-1}^{hh} + M_{t-1}^{hR} + M_{t-1}^{RR}}$$

For each of these equations, the first term accounts for survival of adult females having the given mated genotype from one day to the next, and the second term accounts for transformation of pupae of the given female genotype into adults. The second term is then multiplied by the fraction of the adult male population having either genotype HH, Hh, HR, hh, hR or RR, depending on the female mated genotype.

Using these equations, we can derive several basic properties of the population, such as the non-zero equilibrium densities of larvae and adults, and the generational or daily mosquito population growth rate in the absence of genetic control. The generational population growth rate, R_M, is equal to the rate of female egg production multiplied by the life expectancy of an adult mosquito multiplied by the proportion of eggs that will survive through all of the juvenile life stages in the absence of density-dependence. This is given by,

$$R_M = \frac{\beta \theta_E \theta_L \theta_P (1-\mu_M)}{2\mu_M} \ . \tag{S51}$$

Substituting formulae for the survival probabilities of the juvenile life stages, we have,

$$R_M = \frac{\beta(1-\mu_L)^{T_E+T_L+T_P}(1-\mu_M)}{2\mu_M} \quad . \tag{S52}$$

And rearranging the formula to express the mortality rates for the juvenile stages as a function of the per-generation mosquito population growth rate, we have,

$$\mu_L = 1-\left(\frac{2R_M\mu_M}{\beta(1-\mu_M)}\right)^{1/(T_E+T_L+T_P)} \quad . \tag{S53}$$

Following Deredec *et al.* (2011), we estimate the daily population growth rate of *An. gambiae* to be 1.096 per day, based on the average rate of population growth at the beginning of three wet seasons in the village of Kwaru, Nigeria, measured as part of the Garki Project (Molineaux & Gramiccia, 1980). Calculating the generation time of *An. gambiae* as $T_E + T_L + T_P + 1/\mu_M = 24.1$ days, this corresponds to a population growth rate of $1.096^{24.1} = 9.1$ per generation. We adopt this value of the population growth rate for the majority of our simulations; but consider values of R_M = 2, 6 and 12 per generation to represent low, medium and high growth rates. In each case, the corresponding value of μ_L is calculated according to Equation S53 and the parameter values in Table S1.

The equilibrium population densities can then be calculated by setting the population densities to be equal across generations in Equations S1, S12, S30 and S48. This leads to the following non-zero equilibria:

$$L_{eq} = \alpha(R_M - 1), \tag{S54}$$

$$N = \frac{2}{\beta\theta_E}\left(\frac{1-\sqrt[T_L]{\theta_L / R_M}}{1-(\theta_L / R_M)}\right)\alpha(R_M - 1). \tag{S55}$$

Here, L_{eq} and N represent the total population equilibria (i.e. $L_{eq} = L_{eq}^{hh}$ and $N = M_{eq}^{hh} + M_{eq}^{hh,hh}$).

These formulations guide the parameter choices, taken from Deredec *et al.* (2011), as shown in Table S1:

Table S1: Parameter values for stochastic, discrete-time model.

Symbol:	Parameter:	Value:	References:
Primary parameters:			
β	Egg production per wild-type female	32 /day	Depinay *et al.* (2004)
T_E	Duration of egg stage	1 day	Depinay *et al.* (2004)
T_L	Duration of larval stage	14 days	Depinay *et al.* (2004)
T_P	Duration of pupal stage	1 day	Depinay *et al.* (2004)
μ_M	Death rate of adult stage	0.123 /day	Molineaux & Gramiccia (1980)
Variable parameters:			
R_M	Per-generation mosquito population growth rate	R_M = 2, 6, 9.1, 12	Molineaux & Gramiccia (1980)
e	Homing rate	0.97 < e < 0.9999	Hammond *et al.* (2016)
ρ	Resistant allele generation rate	$10^{-8} < \rho < 10^{-2}$	Hammond *et al.* (2016)
s	Fertility cost to Hh females	s = 0, 0.907	Hammond *et al.* (2016)
N	Equilibrium adult mosquito population size (male and female)	$10^3 < N < 10^{10}$	

Modeling gRNA multiplexing:

The results depicted in Figure 3 in the main text suggest that the probability of population elimination is independent of the homing rate for e > 98%, but is highly dependent on the resistant allele generation rate, ρ. We propose gRNA multiplexing as a method to reduce the effective resistant allele generation rate and, here, describe how the effective resistant allele generation rate varies with multiplex number.

Multiplex number of two:

For the example of two multiplexed gRNAs, there are two sites within a composite allele at which the gRNAs cleave, both of which may either have the homing construct, H, be resistant to homing, R, or be wild-type, h. We denote the composite allele as {*xy*}, where *x* denotes the first site in the composite allele, *y* denotes the second side in the composite allele, and $x, y \in \{H, R, h\}$.

As multiplexing provides multiple opportunities for homing to occur, we consider a composite allele to have the homing phenotype (the ability to cleave and home into the homologous chromosome at multiple target sites) if at least one of its sites has a functional copy of the homing allele (i.e. the composite alleles {HH}, {Hh} and {HR}). We consider a composite allele to have the homing-resistant phenotype if all of its sites have a homing-resistant allele (i.e. the composite allele {RR}). All other composite alleles are considered to have the wild-type phenotype, i.e. they don't have the homing phenotype but are still potentially receptive to a homing event. For the two-gRNA system, the effective resistant allele generation rate, $\rho_{m=2}$, is then given by,

$$\rho_{m=2} = \frac{\mathrm{P}\{\mathrm{hh}\}}{\mathrm{P}\{\mathrm{hh}\} + \mathrm{P}\{\mathrm{hR}\}}\rho^2 + \frac{\mathrm{P}\{\mathrm{hR}\}}{\mathrm{P}\{\mathrm{hh}\} + \mathrm{P}\{\mathrm{hR}\}}\rho \ . \qquad \text{(S56)}$$

Here, P{hh} and P{hR} represent the proportion of composite alleles that are {hh} and {hR}, respectively, ρ^2 is the probability of generating a {RR} composite allele from a {hh} composite allele when the composite allele on the opposite chromosome has at least one functional copy of the homing allele, and ρ is the probability of generating an {RR} allele from an {hR} allele under the same circumstances.

The relative proportion of {hh} and {hR} composite alleles can be estimated by considering the flux of composite alleles as they become associated with composite alleles on the opposite chromosome having the homing phenotype. For the two-gRNA system, the flux of {hR} composite alleles is given by,

$$\frac{d\mathrm{P}\{\mathrm{hR}\}}{dt} = 2c\rho(1-e-\rho)\mathrm{P}\{\mathrm{hh}\} - c(e+\rho)\mathrm{P}\{\mathrm{hR}\} \ . \qquad \text{(S57)}$$

Here, c represents the rate at which a given chromosome becomes associated with an opposite chromosome having the homing phenotype. The rate of change of the proportion of chromosomes having the {hR} composite allele is equal to the rate at which {hR} composite alleles are generated from {hh} alleles (i.e. through resistant allele generation at one site and wild-type allele maintenance at the other site) subtracting the rate at which {hR} composite alleles are lost through either homing or resistant allele generation at the remaining wild-type site.

Allele frequencies are constantly in flux as a gene drive system spreads into a population; however, if we assume, to a first approximation, that equilibrium is maintained between {hh} and {hR} composite alleles for a given prevalence of composite alleles having the homing phenotype, then the equilibrium solution to Equation S57 suggests the following ratio of {hR} to {hh} composite alleles:

$$\frac{\mathrm{P}\{\mathrm{hR}\}}{\mathrm{P}\{\mathrm{hh}\}} = \frac{2\rho(1-e-\rho)}{e+\rho} \quad . \tag{S58}$$

Substituting this ratio into Equation S56, the effective resistant allele generation rate for two multiplexed gRNAs, $\rho_{m=2}$, is given by,

$$\rho_{m=2} = \rho^2 \frac{2-e-\rho}{e+\rho+2\rho(1-e-\rho)} \quad . \tag{S59}$$

Substituting our homing efficiency of 98% and resistant allele generation rate of 0.13% into Equation S59, we see that the effective resistant allele generation rate for a multiplex number of two is equal to ρ^2 multiplied by a fraction very close to 1 (1.038). I.e. for the circumstances being studied,

$$\rho_{m=2} \approx \rho^2 \quad . \tag{S60}$$

This is a consequence of most resistant allele generation occurring directly from {hh} composite alleles, since {hR} composite alleles are rarely generated and are frequently converted to alleles having the homing phenotype once they have been formed.

Multiplex number of three:

To check whether the same approximation holds for three multiplexed gRNAs, i.e. that most resistant allele generation occurs directly from {hhh} composite alleles, we calculate the effective resistant allele generation rate using the same framework as described above. We denote the composite allele in this case as {*xyz*}, where $x, y, z \in \{H, R, h\}$. As multiplexing provides multiple opportunities for homing to occur, a composite allele is considered to have the homing phenotype if at least on of its sites has a functional copy of the homing allele, a composite allele is considered to have the homing-resistant phenotype if all of its sites have a homing-resistant allele, and all other composite alleles have the wild-type phenotype.

For the three-gRNA system, the effective resistant allele generation rate, $\rho_{m=3}$, is given by,

$$\rho_{m=3} = \frac{\mathrm{P}\{\mathrm{hhh}\}\rho^3 + \mathrm{P}\{\mathrm{hhR}\}\rho^2 + \mathrm{P}\{\mathrm{hRR}\}\rho}{\mathrm{P}\{\mathrm{hhh}\} + \mathrm{P}\{\mathrm{hhR}\} + \mathrm{P}\{\mathrm{hRR}\}} \quad . \tag{S61}$$

Here, P{hhh}, P{hhR} and P{hRR} represent the proportion of composite alleles that are {hhh}, {hhR} and {hRR}, respectively, and ρ^3, ρ^2 and ρ are the probabilities of generating an {RRR} composite allele from an {hhh}, {hhR} and {hRR} composite allele, respectively, when the corresponding allele has at least one functional copy of the homing allele.

As for the two-gRNA case, the relative proportion of {hhh}, {hhR} and {hRR} composite alleles can be estimated by considering the relative flux of allele genotypes as they become associated with corresponding composite alleles having the homing phenotype. For the three-gRNA system, the flux of the {hhR} composite alleles is given by,

$$\frac{d\mathrm{P}\{\mathrm{hhR}\}}{dt} = 3c\rho(1-e-\rho)^2\mathrm{P}\{\mathrm{hhh}\} - c(1-(1-e+\rho)^2)\mathrm{P}\{\mathrm{hhR}\} \quad . \tag{S62}$$

Here, the rate of change of the proportion of chromosomes having the {hhR} composite allele is equal to the rate at which {hhR} composite alleles are generated from {hhh} composite alleles (i.e. through resistant allele formation at one site and wild-type allele maintenance at the other two sites) subtracting the rate at which {hhR} composite alleles are lost through either homing or resistant allele generation at the remaining two wild-type sites. Assuming, to a first

approximation, that equilibrium is maintained between {hhh} and {hhR} composite alleles for a given prevalence of composite alleles having the homing phenotype, then the equilibrium solution to Equation S62 suggests the following ratio of {hhR} to {hhh} composite alleles:

$$\frac{\mathrm{P}\{\mathrm{hhR}\}}{\mathrm{P}\{\mathrm{hhh}\}}=\frac{3\rho(1-e-\rho)^2}{1-(1-e-\rho)^2} \ . \tag{S63}$$

Similarly, the flux of {hRR} composite alleles is given by,

$$\frac{d\mathrm{P}\{\mathrm{hRR}\}}{dt}=3c\rho^2(1-e-\rho)\mathrm{P}\{\mathrm{hhh}\}+2c\rho(1-e+\rho)\mathrm{P}\{\mathrm{hhR}\}-c(e+\rho)\mathrm{P}\{\mathrm{hRR}\} \ . \tag{S64}$$

Here, the rate of change of the proportion of chromosomes having the {hRR} composite allele is equal to the rate at which {hRR} composite alleles are generated from {hhh} composite alleles (i.e. through resistant allele formation at two sites and wild-type allele maintenance at the other site) added to the rate at which {hRR} composite alleles are generated from {hhR} composite alleles (i.e. through resistant allele formation at one site and wild-type allele maintenance at the other site) subtracting the rate at which {hRR} composite alleles are lost through either homing or resistant allele generation at the remaining wild-type site. Substituting Equation S63 into Equation S64 and assuming, to a first approximation, that equilibrium is maintained between {hhh} and {hRR} alleles for a given prevalence of composite alleles having the homing phenotype, then the equilibrium solution to Equation S64 suggests the following ratio of {hRR} to {hhh} composite alleles:

$$\frac{\mathrm{P}\{\mathrm{hRR}\}}{\mathrm{P}\{\mathrm{hhh}\}}=\frac{3\rho^2(1-e-\rho)(2+e^2-2e(1-\rho)-\rho(2-\rho))}{(2-e-\rho)(e+\rho)^2} . \tag{S65}$$

Substituting the ratios in Equations S63 and S65 into Equation S61, the effective resistant allele generation rate for three multiplexed gRNAs, $\rho_{m=3}$, is given by,

$$\rho_{m=3}=\rho^3\frac{(2-e-\rho)(3+e^2-\rho(3-\rho)-e(3-2\rho))}{\begin{pmatrix}e^2(2-9\rho(1-\rho)^2)-e^3(1-3\rho(1-\rho))\\+\rho^2(11-\rho(19-3\rho(4-\rho)))+\rho e(7-9\rho(3-\rho(3-\rho)))\end{pmatrix}} \ . \tag{S66}$$

Substituting our homing efficiency of 98% and resistant allele generation rate of 0.13% into Equation S66, we see that the effective resistant allele generation rate for a multiplex number of three is equal to ρ^3 multiplied by a fraction very close to 1 (1.058). I.e. for the circumstances being studied,

$$\rho_{m=3} \approx \rho^3 . \qquad \text{(S67)}$$

This is a consequence of most resistant allele generation occurring directly from {hhh} composite alleles, since {hhR} and {hRR} composite alleles are rarely generated and are frequently converted to composite alleles having the homing phenotype once they have been formed. We have reason to believe this trend will continue for higher multiplex numbers for the parameter ranges we are exploring here. Therefore, for the purposes of this paper, we will approximate the effective resistant allele generation rate for a multiplex number of m as ρ^m.

<u>Multiplex number of two (reduced homing rate for second gRNA):</u>

In the above calculations, we have ignored the reduced cleavage rate observed for the RGR multiplexing approach in *D. melanogaster* for the second gRNA. If we assume a fractional reduction in cleavage rate, f, and that this will reduce both the homing and resistant allele generation rates by the same amount for the second gRNA, then we can derive how this effects the effective resistant allele generation rate by modifying the previous analysis for two multiplexed gRNAs. We will now consider ordered composite alleles, where {hR} represents a composite allele with a wild-type allele at the first site and a homing-resistant allele at the second site, and {Rh} represents a composite allele with a homing-resistant allele at the first site and a wild-type allele at the second site. For the two-gRNA system, the effective resistant allele generation rate, $\rho_{m=2}$, is now given by,

$$\rho_{m=2} = \frac{\mathrm{P}\{\mathrm{hh}\} f \rho^2 + \mathrm{P}\{\mathrm{Rh}\} f \rho + \mathrm{P}\{\mathrm{hR}\} \rho}{\mathrm{P}\{\mathrm{hh}\} + \mathrm{P}\{\mathrm{Rh}\} + \mathrm{P}\{\mathrm{hR}\}} . \qquad \text{(S68)}$$

Here, $f\rho^2$ is the probability of generating a {RR} composite allele from a {hh} composite allele when the composite allele on the opposite chromosome has at least one functional copy of the homing allele, ρ is the probability of generating a {RR} composite allele from a {hR} composite allele under the same circumstances, and $f\rho$ is the probability of generating a {RR} composite allele from a {Rh} composite allele, where the reduction is due to the reduced cleavage rate at

the second site.

The relative proportions of {hh}, {Rh} and {hR} composite alleles can be estimated by considering the flux of composite alleles as they become associated with composite alleles on the opposite chromosome having the homing phenotype. For the two-gRNA system with reduced cleavage rate at site two, these are given by,

$$\frac{d\mathrm{P}\{\mathrm{Rh}\}}{dt} = c\rho(1-f(e+\rho))\mathrm{P}\{\mathrm{hh}\} - cf(e+\rho)\mathrm{P}\{\mathrm{Rh}\} \ . \quad \text{(S69)}$$

$$\frac{d\mathrm{P}\{\mathrm{hR}\}}{dt} = cf\rho(1-e-\rho)\mathrm{P}\{\mathrm{hh}\} - c(e+\rho)\mathrm{P}\{\mathrm{hR}\} \ . \quad \text{(S70)}$$

Here, {Rh} composite alleles are generated at a faster rate due to the higher likelihood that the second site will remain wild-type, and {hR} composite alleles are generated at a slower rate due to the smaller likelihood that cleavage and hence resistant alleles will occur at the second site. {Rh} composite alleles are also lost at a slower rate due to homing and resistant allele generation occurring at a slower rate at the second site.

If we assume, to a first approximation, that equilibrium is maintained between {hh}, {Rh} and {hR} composite alleles for a given prevalence of composite alleles having the homing phenotype, then the equilibrium solution to Equations S69-S70 suggests the following ratio of {Rh} and {hR} to {hh} composite alleles:

$$\frac{\mathrm{P}\{\mathrm{Rh}\}}{\mathrm{P}\{\mathrm{hh}\}} = \frac{\rho(1-f(e+\rho))}{f(e+\rho)} \ . \quad \text{(S71)}$$

$$\frac{\mathrm{P}\{\mathrm{hR}\}}{\mathrm{P}\{\mathrm{hh}\}} = \frac{f\rho(1-e-\rho)}{e+\rho} \ . \quad \text{(S72)}$$

Substituting these ratios into Equation S68, the effective resistant allele generation rate for two multiplexed gRNAs, $\rho_{m=2}$, with reduced cleavage at site two is given by,

$$\rho_{m=2} = f\rho^2 \frac{1+f(1-e-\rho)}{\rho + f(\rho(1+f)(1-\rho) + e(1-\rho(1+f)))} \ . \quad \text{(S73)}$$

Substituting our homing efficiency of 98% and baseline ρ value of 0.13% into Equation S73, we

see that, for a reduced cleavage rate of 75% at site two, the effective resistant allele generation rate is equal to ρ^2 multiplied by 1.033, and for a reduced cleavage rate of 50% at site two, the effective resistant allele generation rate is ρ^2 multiplied by a 1.027. In fact, even for a drastically reduced cleavage rate of 25% at site two, the effective resistant allele generation rate is still approximately ρ^2 (ρ^2 multiplied by a 1.020). Thus, interestingly, a reduction in cleavage rate at site two doesn't significantly alter the effective resistant allele generation rate, and in fact slightly reduces the rate as compared to that without a reduced cleavage rate at the second site (which was ρ^2 multiplied by 1.038).

Table S2: Primer sequences Sequences

Primer name	Primer sequence, 5' to 3'	Source
OA16-1	TAGCGGATCCGGGAATTGGGAATTGGGCAATATTTAAATGGCGGCCGCGCGCAGATCGCCGATG	Drosophila genomic DNA
OA16-2	TTCGTCCTCACGGGACTCATCAGGGCGATGGCGCGCCTCTGCGGGTCAAAATAGAGATGT	
OA16-3	TCGGCATGGCGAATGGGACAGATCTTTGTGAAGGAACCTTACTTCTGTG	pMos-3xP3-DsRed-attp (addgene plasmid #52904)
OA16-4	GGATCTCTAGAGGTACCGTTGCGGCCGAATTCTTAATTAATGGAACCAGACATGATAAGA	
OA16-5	CTATTTTGACCCGCAGAGGCGCGCCATCGCCCTGATGAGTCCCGTGAGGACGAAACGAGTAAGCTCGTCGGCGATACTTGGATGCCCTGGTTTTAGAGCTAGAAATAGCAAGTTAA	Self annealing primers
OA16-6	TGTTGCCCAGCCGGCGCCAGCGAGGAGGCTGGGACCATGCCGGCCAAAAGCACCGACTCGGTGCCACTTTTTCAAGTTGATAACGGACTAGCCTTATTTTAACTTGCTATTTCTA	
OA16-7	ATCAGGGTTTTTGTCCCATTCGCCATGCCGAAGCATGTTGCCCAGCCGGCGCCAGCGAGG	
OA16-8	CTATTTTGACCCGCAGAGGCGCGCCATCGCCCTGATGAGTCCCGTGAGGACGAAACGAGTAAGCTCGTCGGCGATACTTGGATGCCCTGGTTTTAGAGCTAGAAATAGCAAGTTAA	Self annealing primers
OA16-9	CGGCATGGCGAATGGGACAAAAACCCTGATGAGTCCCGTGAGGACGAAACGAGTAAGCTCGTCGGTTTTGGACACTGGAACCGGTTTTAGAGCTAGAAATAGCAAGTTAA	
OA16-10	GGTTCCTTCACAAAGATCTGTCCCATTCGCCATGCCGAAGCATGTTGCCCAGCCGGCGCC	

OA16-S1<br>OA16-S2<br>OA16-S3<br>OA16-S4	CCTATCCGGGCGAACTTTTG<br>AAGAGGTCATCCTGCTGGAC<br>TCAAATACAGCTGGAGATTG<br>TATCCGTGGTCAAGTCAAAG	Sequencing primers for *white* (S1 and S2) and *yellow* (S3 and S4) loci

References:

1. Deredec A, Godfray HCJ, Burt A (2011) Requirements for effective malaria control with homing endonuclease genes. *Proc Natl Acad Sci U S A* 108(43):E874–80.

2. Depinay J-MO, et al. (2004) A simulation model of African Anopheles ecology and population dynamics for the analysis of malaria transmission. *Malar J* 3:29.

3. Molineaux L, Gramiccia G, Organization WH (1980) *The Garki Project: Research on the Epidemiology and Control of Malaria in the Sudan Savanna of West Africa* (World Health Organization).

4. Hammond A, et al. (2016) A CRISPR-Cas9 gene drive system targeting female reproduction in the malaria mosquito vector Anopheles gambiae. *Nat Biotechnol* 34(1):78–83.
